# Insect I‐Type Lysozymes Function as Antiviral Proteases by Forming Biomolecular Condensates

**DOI:** 10.1002/advs.202514408

**Published:** 2025-10-27

**Authors:** Yu Du, Yuqing Xiao, Manman Hu, Jinhua Yang, You Li, Taiyun Wei

**Affiliations:** ^1^ State Key Laboratory of Agricultural and Forestry Biosecurity Fujian Agriculture and Forestry University Fuzhou Fujian 350002 China

**Keywords:** antiviral protease, liquid–liquid phase separation, lysozyme, rice viruses, Toll signaling pathway

## Abstract

Lysozymes are well‐known for their ability to cleave bacterial peptidoglycan, but their potential to hydrolyze viral components as a form of antiviral defense remains poorly understood. This study demonstrates that insect i‐type lysozymes (Lyz‐I1), regulated by the Toll signaling pathway, function as proteases that directly cleave viral proteins. Structural and functional analyses reveal that the catalytic dyad Glu34/Asp50 in leafhopper Lyz‐I1, while retaining its essential role in bacterial peptidoglycan hydrolysis, also mediates specific binding to Lys180 on the viroplasm protein Pns9 of rice gall dwarf virus (RGDV). This interaction catalyzes the cleavage of the adjacent peptide bond of Lys180, leading to Pns9 degradation, which disrupts viroplasm assembly and inhibits viral replication. Notably, this proteolytic antiviral mechanism of Lyz‐I1 shows evolutionary conservation across major rice reoviruses and their respective leafhopper or planthopper vectors. Additionally, leafhopper Lyz‐I1 undergoes liquid–liquid phase separation, forming biomolecular condensates that concentrate Pns9 and enhance proteolytic efficiency. Critically, exogenous application of Lyz‐I1 not only effectively reduces viral titer and disease symptoms in RGDV‐infected rice plants but also induces plant immune defense. Consequently, this work provides the evidence that lysozymes can function as specific antiviral proteases, establishing a foundation for innovative control strategies against viral diseases.

## Graphical abstract

Upon rice viral infection, the insect vector activates the Toll–MyD88–Dorsal signaling cascade, inducing i‐type lysozyme (Lyz‐I1) expression. Lyz‐I1 functions as an antiviral protease through its conserved catalytic dyad Glu/Asp (E/D), mediating cleavage of viral proteins at specific Lys (K) residues. This site‐specific proteolysis disrupts essential viral protein functions, thereby inhibiting viral replication.

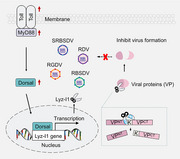

## 1 Introduction

Lysozymes, ubiquitously distributed across the animal and plant kingdoms, constitute a cornerstone of innate immunity.^[^ ^1^ ^]^ Originally identified nearly a century ago by Alexander Fleming for their bacteriolytic activity,^[^ ^2^ ^]^ these enzymes are primarily classified into several types, with c‐type (chicken or conventional) and i‐type (invertebrate) being the most extensively studied.^[^ ^3^ ^]^ Their defining characteristic is the enzymatic hydrolysis of β‐1,4‐glycosidic linkages between *N*‐acetylmuramic acid (NAM) and *N*‐acetylglucosamine (NAG) within bacterial peptidoglycan.^[^ ^1^, ^4^ ^]^ This activity, mediated by a conserved catalytic dyad (typically Glu and Asp residues in c‐type lysozymes), constitutes a well‐established defense mechanism against bacterial pathogens.^[^ ^5^ ^]^ In stark contrast, the role of lysozymes in antiviral defense remains poorly characterized. While some studies describe lysozymes’ antiviral effects in medical applications,^[^ ^6^ ^]^ their underlying molecular mechanisms remain poorly understood. Proposed nonenzymatic antiviral actions of lysozymes encompass direct virion aggregation or neutralization,^[^ ^7^ ^]^ potentially mediated through cationic interactions with viral envelopes or capsids,^[^ ^8^ ^]^ or indirect immunomodulatory effects.^[^ ^9^ ^]^ Critically, a fundamental unresolved question is whether lysozymes can act as hydrolytic enzymes against viral components, analogous to their cleavage of peptidoglycan but targeting viral substrates.

Insects, lacking the adaptive immunity of vertebrates, primarily rely on robust innate immune mechanisms for defense against pathogens.^[^ ^10^ ^]^ Central to this system is the production of diverse antimicrobial peptides (AMPs).^[^ ^11^ ^]^ These compact, typically cationic peptides are rapidly synthesized upon pathogen invasion and exhibit broad‐spectrum activity against diverse pathogens.^[^ ^12^ ^]^ Major AMP families in insects include defensins,^[^ ^13^ ^]^ cecropins,^[^ ^14^ ^]^ proline‐rich AMPs,^[^ ^15^ ^]^ and the functionally significant lysozymes.^[^ ^16^ ^]^ Insect lysozymes, primarily classified as c‐type and i‐type, are established as key immune effector molecules upregulated during infection, and are widely expressed across various tissues, particularly in the fat body, midgut, and hemocytes.^[^ ^17^ ^]^ Within the canonical framework of insect immunity, their primary role is antibacterial, mediated through peptidoglycan hydrolysis.^[^ ^22^ ^]^ Expression of lysozyme genes is regulated by conserved immune signaling cascades, notably the Toll and Imd pathways.^[^ ^18^ ^]^ Receptors upstream of these cascades recognize pathogen‐associated molecular patterns (PAMPs), transducing signals that activate NF‐κB transcription factor homologs (e.g., Dorsal, Relish), which directly drive AMP genes transcription.^[^ ^19^ ^]^ As immune effectors, lysozymes also contribute to antiviral defense in insects. For example, in the silkworm *Bombyx mori*, lysozyme expression is strongly upregulated in hemocytes following infection with *Bombyx mori* nucleopolyhedrovirus and has been demonstrated to exert antiviral effects.^[^ ^20^ ^]^ Similarly, in the mosquito *Anopheles gambiae*, two lysozymes mediate specific antiviral immune responses against systemic O'nyong‐nyong virus infection.^[^ ^21^ ^]^ Although insect lysozymes participate in antiviral defense, their precise antiviral mechanisms remain unclear. Current evidence suggests that the observed effects arise exclusively from indirect actions or nonenzymatic properties.

This study resolves this critical knowledge gap by demonstrating a direct enzymatic antiviral mechanism mediated through functional repurposing of an insect lysozyme. Approximately 80% of plant viruses depend on transmission by sap‐sucking insect vectors, including planthoppers, leafhoppers, and aphids.^[^ ^22^ ^]^ Devastating agricultural epidemics across Asia over recent decades have been caused by leafhopper‐ or planthopper‐transmitted plant reoviruses, including rice gall dwarf virus (RGDV), rice dwarf virus (RDV), rice black‐streaked dwarf virus (RBSDV), and southern rice black‐streaked dwarf virus (SRBSDV).^[^ ^23^ ^]^ Here, we identify an i‐type lysozyme (Lyz‐I1) in leafhopper *Recilia dorsalis*, regulated by the Toll signaling pathway, as a potent and specific antiviral factor against RGDV infection. Crucially, we demonstrate that its antiviral activity relies on a previously unrecognized proteolytic capacity for cleaving an essential viral replication protein. Molecular characterization reveals that the Lyz‐I1 catalytic dyad, conserved for its classic role in peptidoglycan hydrolysis, is functionally repurposed for viral protein cleavage. Remarkably, this proteolytic antiviral mechanism shows evolutionary conservation across major rice reoviruses and their respective insect vectors. Consequently, this work provides the first definitive evidence that lysozymes can function as specific antiviral proteases, establishing a foundation for innovative control strategies against arthropod‐borne plant viral diseases.

## 2 Results

### 2.1 Identification of RdLyz‐I1 as a Key Antiviral Lysozyme that Specifically Interacts with RGDV Pns9

**Figure 1:**
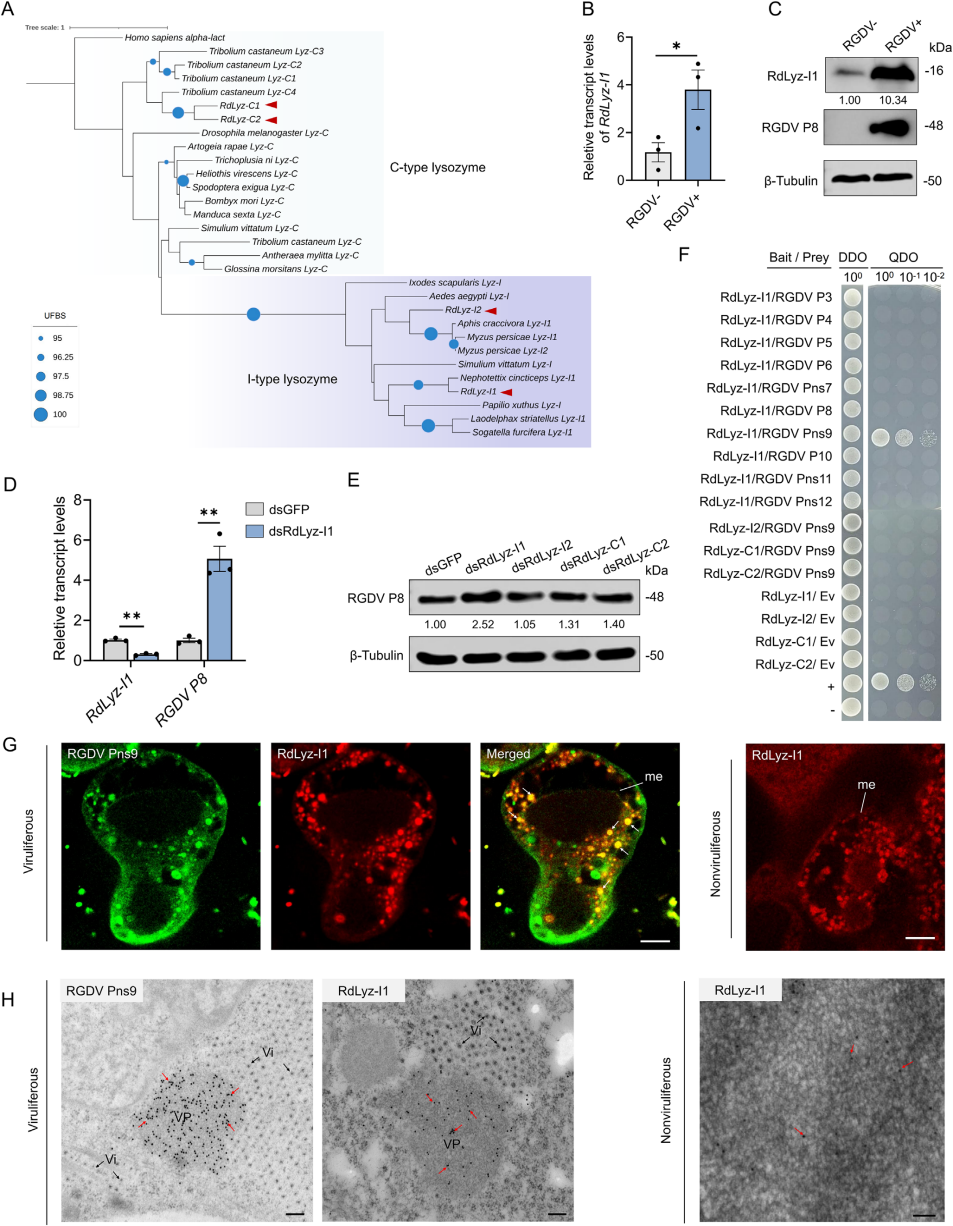
RdLyz‐I1 interacts with RGDV Pns9 and inhibits viral propagation in insect vectors. A) Phylogeny of insect lysozymes. Maximum likelihood phylogenetic tree constructed with RAxML using amino acid sequences of insect lysozymes, with human α‐lactalbumin as the outgroup. Red arrows highlight four *R. dorsalis* lysozymes. B) RT‐qPCR analysis showing the expression of *RdLyz‐I1* in nonviruliferous or viruliferous leafhoppers. RNA was extracted from 30 pooled leafhoppers per replicate. C) Western blot analysis showing RdLyz‐I1 protein accumulation levels in nonviruliferous or viruliferous leafhoppers. Protein was extracted from 30 pooled leafhoppers per replicate. Relative band intensities for RdLyz‐I1 levels are shown below. D) Effects of *RdLyz‐I1* knockdown on RGDV infection, as determined by RT‐qPCR. RNA was extracted from 30 pooled viruliferous leafhoppers per replicate. Relative expression for *RdLyz‐I1* and RGDV *P8* are shown. E) Effects of *RdLyz‐I1*, *RdLyz‐I2*, *RdLyz‐C1*, and *RdLyz‐C2* knockdown on RGDV infection, as determined by western blot. Protein was extracted from 30 pooled RGDV‐positive leafhoppers per replicate. Relative band intensities for RGDV P8 are shown below. F) Y2H analysis showing the interactions between viral proteins and lysozymes. Transformants were plated on DDO and QDO. DDO, SD/‐Trp–Leu medium, QDO, SD/‐Trp–Leu–His–Ade medium. Data represent one typical result from three independent experiments. G) Immunofluorescence microscopy showing the colocalization of RdLyz‐I1 with RGDV Pns9 in virus‐infected midgut. Midguts from viruliferous and nonviruliferous insects were immunolabeled with RGDV Pns9‐FITC (green) or RdLyz‐I1‐rhodamine (red). me, midgut epithelium; white arrows indicate the sites colocalized by RdLyz‐I1 and RGDV Pns9. Scale bars: 5 µm. H) Immunogold labeling of RdLyz‐I1 within RGDV Pns9‐formed structures in virus‐infected midgut tissues. Midguts from viruliferous and nonviruliferous insects were subjected to immunolabeling using RdLyz‐I1 or RGDV Pns9 antibodies as the primary antibody, followed by treatment with 10 nm gold particle‐conjugated IgG as the secondary antibody. Red arrows indicate gold particles. Vp, viroplasm; Vi, virions. Bars, 100 µm. (B,D) Data are presented as mean ± SEM. *, *p* < 0.05; **, *p* < 0.01 (Student’ s *t*‐test). (C,E) β‐Tubulin bands indicate equal protein loading. All data represent three biological replicates.

Bioinformatics analysis of *R. dorsalis* identified four *lysozyme* genes, including two c‐type (*RdLyz‐C1* and *RdLyz‐C2*) and two i‐type (*RdLyz‐I1* and *RdLyz‐I2*) *lysozymes* (**Figure** **1**A). RT‐qPCR assays showed that RGDV infection increased the transcript levels of *RdLyz‐C1* and *RdLyz‐I1* in viruliferous leafhopper at 6 days post‐first access to diseased plants (padp), with *RdLyz‐I1* showing the highest expression (Figure 1B; Figure S1A,B, Supporting Information). Protein levels of RdLyz‐I1 were consistent with the RT‐qPCR results (Figure 1C). Functional assays revealed that knockdown of *RdLyz‐I1* expression via microinjection of in vitro synthesized dsRNAs targeting *RdLyz‐I1* (dsRdLyz‐I1) significantly increased the accumulation of RGDV major outer capsid protein P8 (Figure 1D,E). In contrast, knockdown of the other three *lysozymes* had no significant effect on viral accumulation (Figure 1E; Figure S1C–E, Supporting Information), indicating that RdLyz‐I1 serves as the primary antiviral lysozyme in leafhoppers.

The RGDV genome encodes six structural and six nonstructural proteins.^[^ ^24^ ^]^ Among these, nonstructural proteins Pns7, Pns9, and Pns12 form the initial viroplasm matrix, which serves as the site for viral replication and progeny virion assembly.^[^ ^25^ ^]^ Importantly, Pns9 is essential for viroplasm inclusion formation during RGDV infection.^[^ ^26^ ^]^ To elucidate RdLyz‐I1's antiviral mechanism, we performed yeast two‐hybrid (Y2H) screening using RdLyz‐I1 as bait, identifying a specific interaction with RGDV Pns9 but not with other tested viral proteins (Figure 1F). This interaction was further validated by glutathione S‐transferase (GST) pull‐down assays (Figure S1F, Supporting Information). No direct interaction was observed between the other three leafhopper lysozymes with RGDV Pns9 (Figure 1F; Figure S1F, Supporting Information). At 6 days padp, immunofluorescence microscopy of virus‐infected midguts of leafhopper showed the extensive colocalization of RdLyz‐I1 with RGDV viroplasm of Pns9 (Figure 1G). Immunoelectron microscopy further confirmed that RGDV Pns9 antibodies densely reacted with RGDV viroplasm matrices within virus‐infected leafhopper midgut cells (Figure 1H). Significantly, the Lyz‐I1 antibody also specifically reacted with these viroplasm matrices (Figure 1H), suggesting a potential role for RdLyz‐I1 in targeting viral replication factories to inhibit RGDV infection.

### 2.2 Residues Lys180‐Dependent Cleavage of RGDV Pns9 by RdLyz‐I1

**Figure 2:**
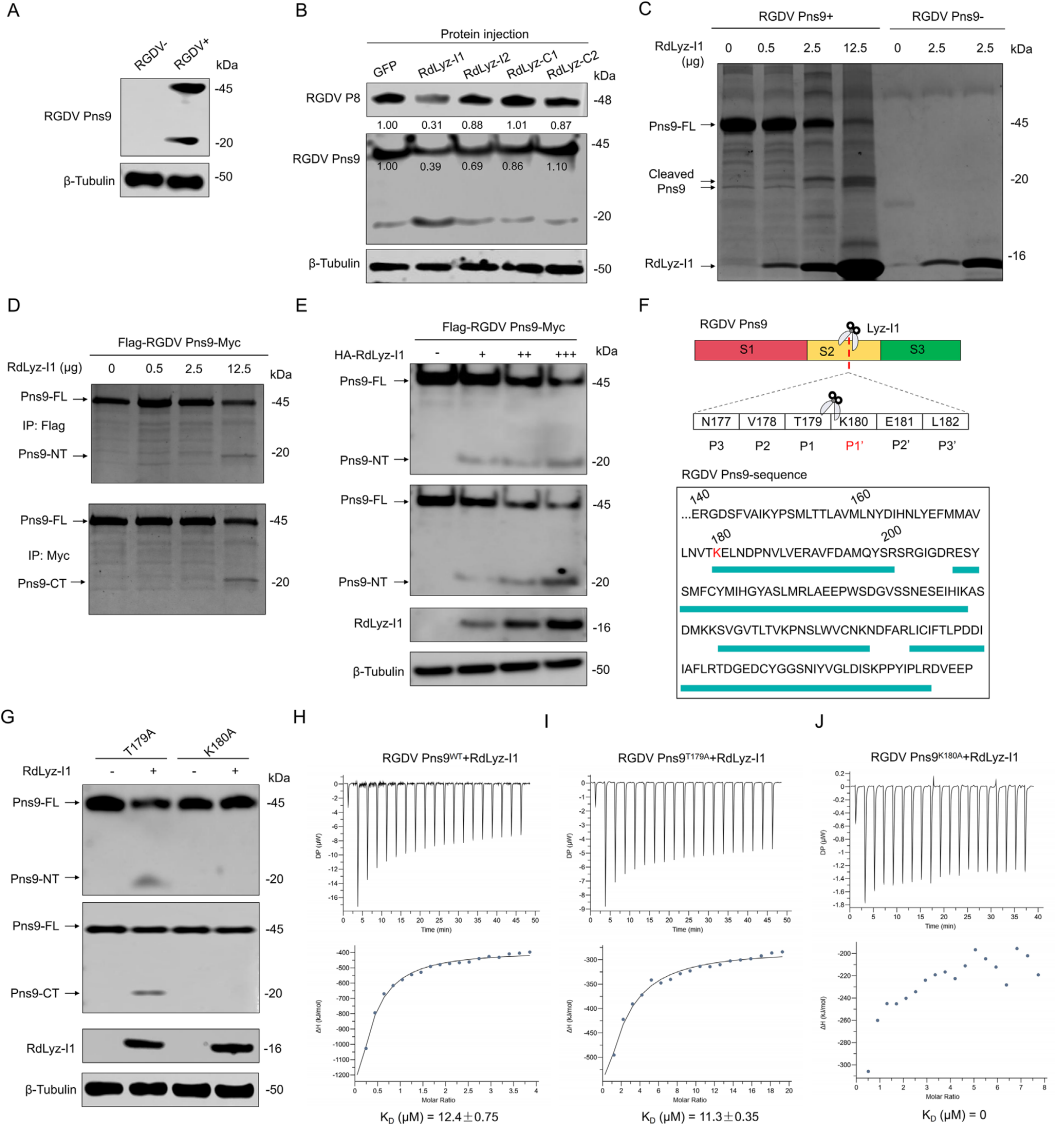
RdLyz‐I1 acts as a protease by cleaving RGDV Pns9 into two fragments. A) Western blot analysis showing RGDV Pns9 protein accumulation level in nonviruliferous or viruliferous leafhoppers. Protein was extracted from 30 pooled leafhoppers per replicate. B) Western blot analysis showing RGDV P8 and RGDV Pns9 accumulation levels in viruliferous leafhoppers treated with purified RdLyz‐I1, RdLyz‐I2, RdLyz‐C1, or RdLyz‐C2 protein (1 µg µL^−1^) for 24 h. GFP protein served as a control. Protein was extracted from 30 pooled leafhoppers per replicate. Relative band intensities are shown below. C) SDS‐PAGE analysis of in vitro cleavage of 10 µg Flag–RGDV Pns9–Myc recombinant protein with different amounts of RdLyz‐I1‐purified recombinant protein (0, 0.5, 2.5, and 12.5 µg) for 2 h at 37 °C. Control reactions containing only RdLyz‐I1 protein (0, 0.5, and 2.5 µg) confirmed the absence of nonspecific protein degradation. D) In vitro cleavage of 10 µg Flag–Pns9–Myc recombinant protein with different amounts of RdLyz‐I1‐purified recombinant protein (0, 0.5, 2.5, and 12.5 µg), followed by Co‐IP assays using Flag and Myc antibodies and detection via SDS‐PAGE analysis. E) Western blot analysis of RGDV Pns9 cleavage and RdLyz‐I1 expression in HEK‐293T cells cotransfected for 24 h with 1.0 µg Flag–RGDV Pns9–Myc and different amounts of HA‐RdLyz‐I1 (0, 0.5, 1.0, 2.0 µg) expression plasmids. Proteins were detected using anti‐Myc, anti‐Flag, and anti‐HA antibodies. F) The cleavage site of RGDV Pns9 and protein sequence analysis of RGDV Pns9 C‐terminal fragments cut for mass spectrometry identification. Unique peptides detected by mass spectrometry beyond the K180 cleavage site are highlighted within green rectangles. G) Western blot analysis of RGDV Pns9 cleavage and RdLyz‐I1 expression in HEK‐293T cells cotransfected for 24 h with 2.0 µg HA‐RdLyz‐I1 plasmids and 1.0 µg mutant Pns9 expression plasmids (Pns9^T179A^ or Pns9^K180A^). Proteins were detected using anti‐Myc, anti‐Flag, and anti‐HA antibodies. H–J) Measurements of dissociation constants (K_D_) for 10 µm RdLyz‐I1‐purified protein binding to 0.5 µm of either RGDV Pns9^WT^ (H), Pns9^T179A^ (I) or Pns9^K180A^ (J) purified protein, determined by ITC analysis to assess binding affinity. (A,B,E,G) β‐Tubulin bands indicate equal protein loading. All data represent three biological replicates.

We next investigated how RdLyz‐I1 exerts antiviral activity through RGDV Pns9 interaction. In viruliferous leafhoppers, we detected the full‐length Pns9 (≈45 kDa) of RGDV and a cleaved 20 kDa fragment (**Figure** **2**A). Microinjection of purified RdLyz‐I1, but not other leafhopper lysozymes, significantly enhanced the cleavage of Pns9 and resulted in reduced accumulation of RGDV P8 (Figure 2B). To test whether RdLyz‐I1 directly cleaves RGDV Pns9, we purified full‐length of RGDV Pns9 (Pns9‐FL) protein with an N‐terminal His‐Flag tag and a C‐terminal Myc tag, along with RdLyz‐I1 containing an N‐terminal His tag and a C‐terminal HA tag for in vitro cleavage assays (Figure S1G, Supporting Information). SDS‐PAGE analysis of coincubation mixtures showed gradual reduction of the RGDV Pns9 band proportional to RdLyz‐I1 concentration, with the appearance of two new ≈20 kDa fragments (Figure 2C), indicating RGDV Pns9 processing into two major fragments. To identify cleavage fragments, we performed co‐immunoprecipitation (Co‐IP) assays using Flag‐ or Myc‐conjugated magnetic beads to detect the cleaved fragments from coincubation mixtures in the in vitro cleavage assay. Results revealed distinct Pns9 cleavage fragments after treatment with 12.5 µg RdLyz‐I1 (Figure 2D), confirming direct cleavage of Pns9 by RdLyz‐I1. Consistent with these findings, coexpressing RdLyz‐I1 with Pns9 in HEK‐293T cells induced Pns9 cleavage (Figure 2E). As c‐type lysozymes typically exert antibacterial function by degrading bacterial peptidoglycan, we investigated whether c‐type lysozyme could cleave Pns9. We constructed HA‐RdLyz‐C1, the C type lysozyme from *R. dorsalis*. While coexpressing RdLyz‐C1 with Pns9 in HEK‐293T cells, we failed to detect distinct cleavage fragments (Figure S2A, Supporting Information), suggesting RdLyz‐I1 specifically cleaves Pns9.

To map the Pns9 region targeted by RdLyz‐I1, we divided Pns9 into three distinct structural domains (S1–S3) based on α‐helices and linker regions. We found that the S1 region comprises a 148‐amino acid (aa) linker region, while the S2 region forms an 80 aa structure featuring four α‐helices, and the S3 region constitutes a 93 aa structure with two α‐helices (Figure S2B, Supporting Information). We further constructed deletion mutants of these Pns9 domains and verified RdLyz‐I1 interactions via Y2H assays. Results revealed that deletion of the S2 region abolished the interaction between Pns9 and RdLyz‐I1, indicating S2 is critical for their binding (Figure S2C, Supporting Information). Accordingly, coexpression of RdLyz‐I1 with Pns9^ΔS1^ or Pns9^ΔS3^, but not Pns9^ΔS2^, in HEK‐293T cells resulted in Pns9 cleavage (Figure S2D–F, Supporting Information), demonstrating RdLyz‐I1 cleaves Pns9 specifically within the S2 region.

To determine the precise cleavage site, we excised the ≈20 kDa C‐terminal fragment from SDS‐PAGE gel and performed trypsin digestion and then performed mass spectrometry analysis. The first identifiable peptide from the cleavage products mapped cleavage between Thr179 (T179) and Lys180 (K180) (Figure 2F). To define the exact position, we mutated residues T179 and K180 to Ala (A). Strikingly, K180A mutant drastically abolished RdLyz‐I1 cleavage in expressing HEK‐293T cells; however, T179A mutant did not affect cleavage (Figure 2G). Isothermal titration calorimetry (ITC) assays were used to assess the binding capacity of RdLyz‐I1 with Pns9 and its mutants. The dissociation constant (K_D_) for Pns9 was 12.4 µm while for Pns9^T179A^ was 11.3 µm (Figure 2H,I); however, Pns9^K180A^ showed no detectable binding (Figure 2J). These results demonstrate that K180 in the S2 region of Pns9 is critical for its binding to RdLyz‐I1, ultimately leading to the cleavage of Pns9 between residues T179 and K180.

### 2.3 Catalytic Dyad Glu34/Asp50 of RdLyz‐I1 Mediates Pns9 Cleavage

**Figure 3:**
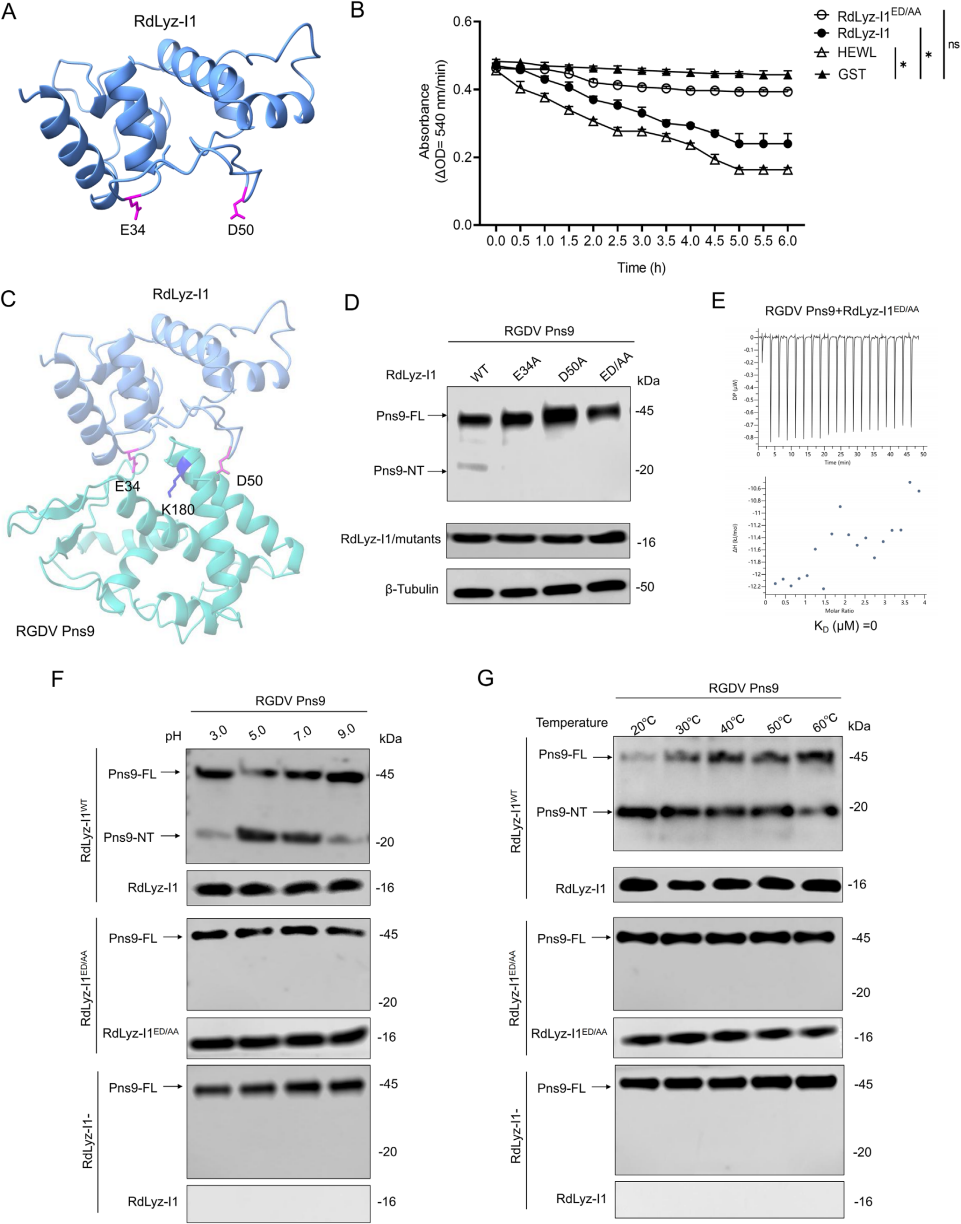
Catalytic dyad Glu34/Asp50 of RdLyz‐I1 mediates RGDV Pns9 cleavage. A) Predicted structure of RdLyz‐I1, highlighting catalytic residues Glu34 (E34) and Asp50 (D50) in purple. B) Amidase activity assay of 10 µg RdLyz‐I1 and RdLyz‐I1^ED/AA^ in vitro. The kinetics of *E. coli* peptidoglycan degradation were measured by recording enzymatic activity at 540 nm every 30 min for 6 h. GST protein served as the negative control, while commercial purified HEWL was used as the positive control. Means (± SEM) are computed from three biological replicates. *, *p* < 0.05; ns, not significant. C) Predicted structure of the RdLyz‐I1–RGDV Pns9 complex using AlphaFold3. RdLyz‐I1 (26–149 aa) is shown in blue, and RGDV Pns9 (151–315 aa) is highlighted in cyan. Key residues are highlighted, including E34 and D50 within RdLyz‐I1, alongside K180 of RGDV Pns9. D) Western blot analysis of RGDV Pns9 cleavage and RdLyz‐I1 expression in HEK‐293T cells transfected for 24 h with 1.0 µg Flag–RGDV Pns9–Myc and 2.0 µg of wild‐type RdLyz‐I1 or mutant variants (RdLyz‐I1^E34A^, RdLyz‐I1^D50A^ or RdLyz‐I1^ED/AA^) expression plasmids. Proteins were detected using anti‐Flag and anti‐HA antibodies. E) Measurements of dissociation constants (K_D_) for 10 µm of RdLyz‐I1^ED/AA^ binding to 0.5 µm RGDV Pns9, determined by ITC analysis to assess binding affinity. F) The effect of pH (3.0, 5.0, 7.0, or 9.0) on the cleavage of 10 µg RGDV Pns9 protein by 10 µg purified RdLyz‐I1 or RdLyz‐I1^ED/AA^ was analyzed via western blot in in vitro assays. Cleavage products and RdLyz‐I1 were detected using anti‐Flag and anti‐HA antibodies. G) The effect of varying temperatures (20, 30, 40, 50 or 60 °C) on the cleavage of 10 µg RGDV Pns9 protein by 10 µg purified RdLyz‐I1 or RdLyz‐I1^ED/AA^ was analyzed via western blot in in vitro assays. Cleavage products and RdLyz‐I1 were detected using anti‐Flag and anti‐HA antibodies. (D) β‐Tubulin bands indicate equal protein loading. All data represent three biological replicates.

Hen egg‐white lysozyme (HEWL), a c‐type lysozyme, excises bacterial peptidoglycan through its catalytic residues Glu35 (E35) and Asp52 (D52) (Figure S3A, Supporting Information).^[^ ^5^ ^]^ To clarify RdLyz‐I1's enzymatic active site, we compared its structure with HEWL. Strikingly, RdLyz‐I1 maintained the characteristic five α‐helices architecture identical to HEWL, with perfect conservation of the catalytic residues E34/D50 (**Figure** **3**A). Additionally, we conducted an amidase activity assay to directly compare the peptidoglycan‐degrading capabilities of HEWL, RdLyz‐I1, and RdLyz‐I1^ED/AA^ (double mutant E34A/D50A) in vitro. HEWL and RdLyz‐I1 possessed similar levels of amidase activity, enabling both enzymes to effectively degrade *Escherichia coli* peptidoglycan, while RdLyz‐I1^ED/AA^ exhibited significantly reduced activity (Figure 3B). Structural modeling further suggested that the catalytic residues E34 and D50 in RdLyz‐I1 were positioned to potentially facilitate peptidoglycan binding (Figure S3B, Supporting Information). These results demonstrate that, like HEWL, RdLyz‐I1 utilizes its conserved E34/D50 residues as a catalytic dyad to hydrolyze glycosidic bonds in bacterial peptidoglycan.

We investigated whether the catalytic dyad E34 and D50 mediate the proteolytic cleavage of Pns9 by RdLyz‐I1. Protein structure prediction and docking analyses modeled the RdLyz‐I1/Pns9 interaction complex, revealing close spatial proximity between RdLyz‐I1 and Pns9 (Figure 3C). Functional validation demonstrated that the RdLyz‐I1 mutants E34A, D50A, and ED/AA lost Pns9 cleavage activity when co–expressed in HEK‐293T cells (Figure 3D). Importantly, ITC assays confirmed that the RdLyz‐I1^ED/AA^ mutant showed no detectable binding to Pns9 (K_D_ = 0 µm) (Figure 3E). These results suggest that RdLyz‐I1 recognizes the K180 residue of Pns9 through its catalytic cdyad E34/D50, cleaving the peptide bond adjacent to the target side chain.

To characterize RdLyz‐I1's enzymatic properties and stability, we systematically evaluated the influence of pH and temperature on its proteolytic activity toward Pns9 using in vitro cleavage assays. First, 12.5 µg recombinant RdLyz‐I1 or RdLyz‐I1^ED/AA^ was preincubated in buffers spanning a pH gradient (3.0–9.0) at 25 °C for 4 h, followed by incubation with 10 µg purified RGDV Pns9 protein. Quantitative analysis of Pns9 cleavage products revealed maximal proteolytic activity at pH 5.0, with negligible activity observed under alkaline conditions (pH 9.0) (Figure 3F). To determine the optimal temperature for RdLyz‐I1 activity, we preincubated the RdLyz‐I1 or RdLyz‐I1^ED/AA^ protein at pH 5.0 across a tested temperature gradient (20–60 °C) for 4 h before substrate addition. RdLyz‐I1 exhibited peak activity at 20 °C, maintaining considerable efficiency across the 20–30 °C range (Figure 3G). No cleavage of RGDV Pns9 was observed with the RdLyz‐I1^ED/AA^ mutant under any condition tested (Figure 3F,G). These results collectively demonstrate that RdLyz‐I1 functions optimally under mildly acidic and cool conditions.

### 2.4 Conservation of Lyz‐I1‐Mediated Viral Protein Cleavage in Insect Vectors

**Figure 4:**
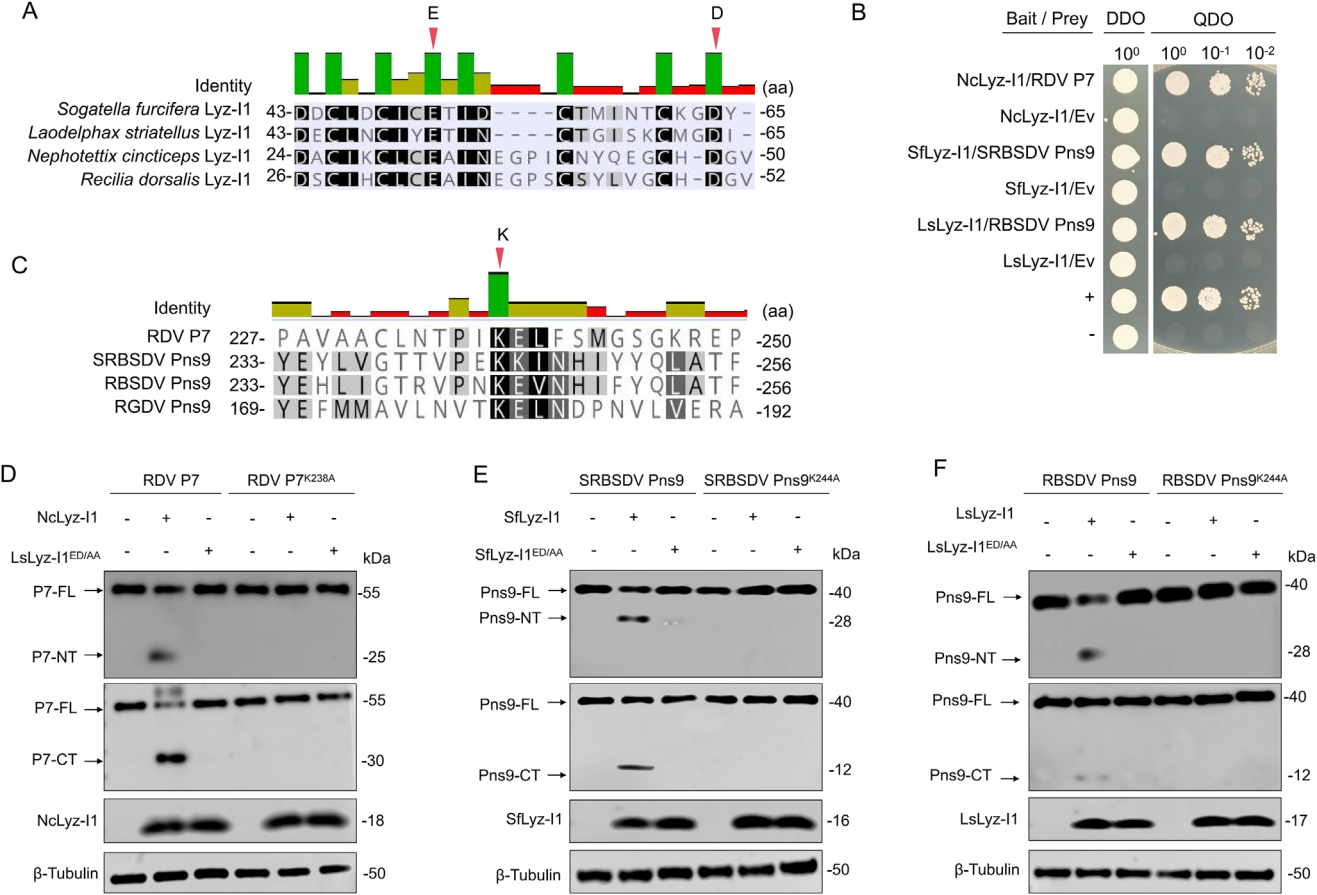
Conservation of Lyz‐I1‐mediated rice viral protein cleavage in insect vectors. A) Sequence alignment of conserved catalytic residues for Lyz‐I1 across different insect vectors (*S. furcifera*, *L. striatellus*, *N. cincticeps*, *R. dorsalis*). The consensus catalytic dyad E/D is marked with red arrows. B) Y2H analysis revealed specific protein–protein interactions between NcLyz‐I1 and RDV P7, SfLyz‐I1 and SRBSDV Pns9, as well as LsLyz‐I1 and RBSDV Pns9. Transformants were plated on DDO (SD/‐Trp–Leu) and QDO (SD/‐Trp–Leu–His–Ade) media. Data represent one typical result from three independent experiments. C) Sequence alignment of conserved cleavage site‐containing regions from rice viral proteins (RDV P7, SRBSDV Pns9, RBSDV Pns9, RGDV Pns9). The consensus residue K is marked with red arrow. D) Western blot analysis of RDV P7 cleavage and NcLyz‐I1 expression in HEK‐293T cells cotransfected for 24 h with 2.0 µg of either HA‐NcLyz‐I1 or HA‐NcLyz‐I1^ED/AA^ alongside 1.0 µg of either Flag–RDV P7–Myc or Flag–RDV P7^K238A^–Myc expression plasmids. Proteins were detected using anti‐Myc, anti‐Flag, and anti‐HA antibodies. E) Western blot analysis of SRBSDV Pns9 cleavage and SfLyz‐I1 expression in HEK‐293T cells cotransfected for 24 h with 2.0 µg of either HA‐SfLyz‐I1 or HA‐SfLyz‐I1^ED/AA^ alongside 1.0 µg of either Flag–SRBSDV Pns9–Myc or Flag–SRBSDV Pns9^K244A^–Myc expression plasmids. Proteins were detected using anti‐Myc, anti‐Flag, and anti‐HA antibodies. F) Western blot analysis of RBSDV Pns9 cleavage and LsLyz‐I1 expression in HEK‐293T cells cotransfected for 24 h with 2.0 µg of either HA‐LsLyz‐I1 or HA‐LsLyz‐I1^ED/AA^ alongside 1.0 µg of either Flag–RBSDV Pns9–Myc or Flag–RBSDV Pns9^K244A^–Myc expression plasmids. Proteins were detected using anti‐Myc, anti‐Flag, and anti‐HA antibodies. (D–F) β‐Tubulin bands indicate equal protein loading. All data represent three biological replicates.

We then investigated the conserved mechanism of insect Lyz‐I1‐mediated viral protein cleavage in diverse vector‐virus systems. RDV, SRBSDV, and RBSDV, which also belong to the rice reoviruses in the family *Reoviridae*, are transmitted in a persistently propagative manner by the leafhopper *Nephotettix cincticeps*,^[^ ^27^ ^]^ the white‐backed planthopper *Sogatella furcifera*,^[^ ^28^ ^]^ and the small brown planthopper *Laodelphax striatellus*,^[^ ^29^ ^]^ respectively. Strikingly, NcLyz‐I1, SfLyz‐I1, and LsLyz‐I1 maintained the characteristic five α‐helices architecture identical to RdLyz‐I with perfect conservation of the catalytic residues E32/D48, E51/D64, and E51/D64, respectively (**Figure** **4**A; Figure S3C–E, Supporting Information). Y2H assays showed that NcLyz‐I1, SfLyz‐I1, and LsLyz‐I1 interacted with the viral replication‐related structural protein P7 of RDV, viroplasm‐related nonstructural protein Pns9 of SRBSDV, and viroplasm‐related nonstructural protein Pns9 of RBSDV, respectively (Figure 4B). Similar to K180 on RGDV Pns9, sequence alignment revealed conserved putative cleavage sites at K238 on RDV P7, and K244 on SRBSDV and RBSDV Pns9, respectively (Figure 4C). These results provide evidence for the conservation of Lyz‐I1‐mediated viral protein cleavage in diverse insect vectors.

To test whether RdLyz‐I1 homologs directly cleave their corresponding viral interaction partners, we expressed SRBSDV Pns9, RBSDV Pns9, and RDV P7 alongside their respective Lyz‐I1 homologs in HEK‐293T cells. Remarkably, each viral protein was cleaved into two fragments through their corresponding Lyz‐I1 homologs (Figure 4D–F). However, the mutation of the respective E32/D48 on NcLyz‐I1, E51/D64 on SfLyz‐I1, and E51/D64 on LsLyz‐I1 to ED/AA failed to cleave the respective viral proteins P7 of RDV, Pns9 of SRBSDV, and Pns9 of RBSDV, respectively (Figure 4D–F). Similarly, the mutation of the respective K238 on RDV P7, K244 on SRBSDV and RBSDV Pns9 to K/A prevented their  cleavage by the corresponding insect Lyz‐I1 proteins (Figure 4D–F). Collectively, these results suggest that insect Lyz‐I1 recognizes the K residue of viral proteins through its catalytic dyad formed by E/D, cleaving the peptide bond adjacent to the target side chain.

### 2.5 RdLyz‐I1 Forms Phase‐Separated Condensates Trapping RGDV Pns9

**Figure 5:**
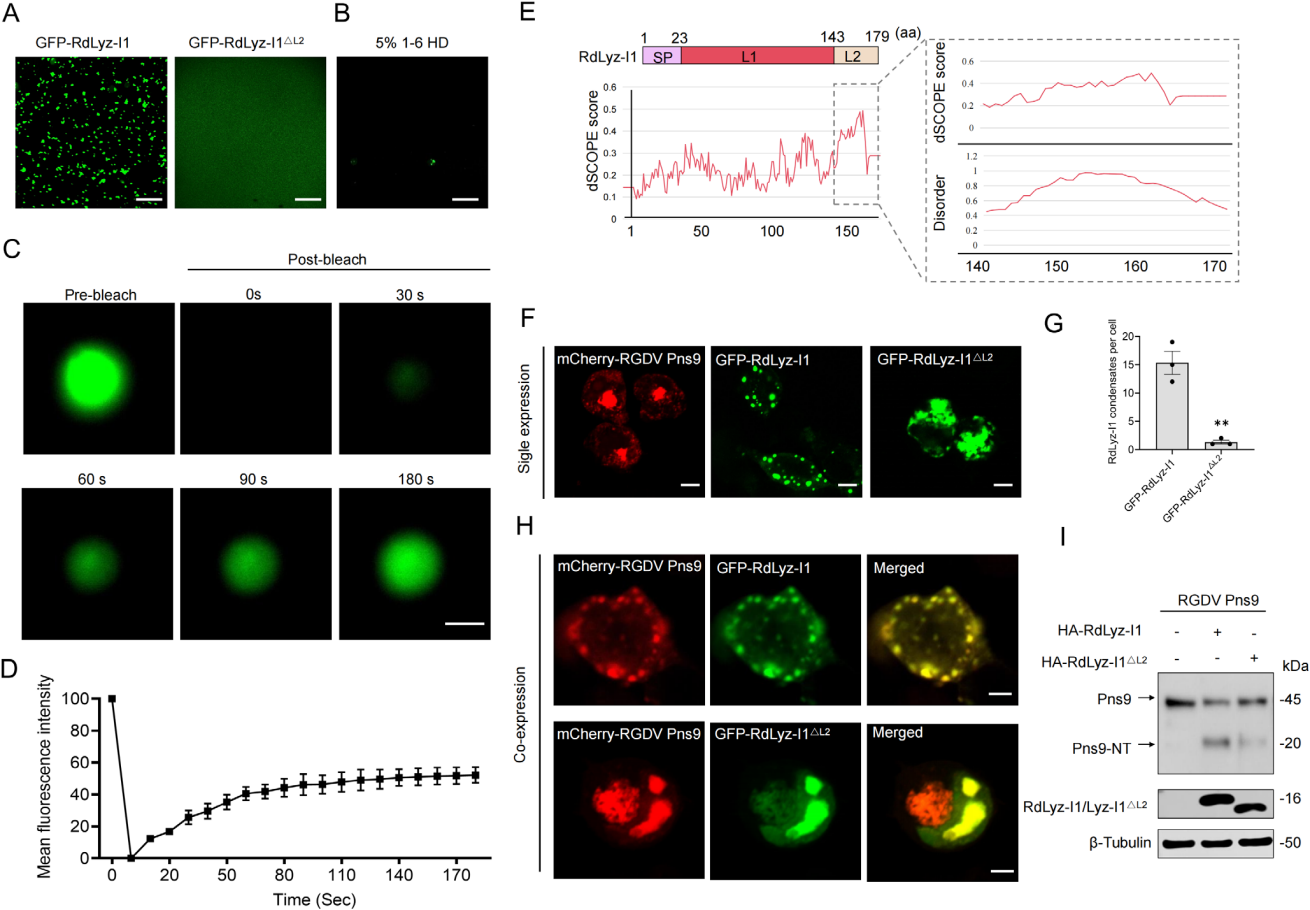
Phase‐separated RdLyz‐I1 condensates recruit RGDV Pns9. A) Confocal microscopy reveals GFP–RdLyz‐I1 forming distinct phase‐separated condensates at 10 µm in buffer containing 150 mm NaCl. In contrast, GFP–RdLyz‐I1^△L2^ exhibits no droplet under identical conditions. Scale bars: 10 µm. B) Confocal microscopy reveals GFP–RdLyz‐I1 droplets formed in vitro at 10 µm in 150 mm NaCl following treatment with 5.0% of 1,6‐HD for 5 min. Scale bars: 10 µm. C) FRAP assay shows recovery within GFP–RdLyz‐I1 droplets formed in vitro at 10 µm in 150 mm NaCl. Scale bars: 10 µm. D) FRAP recovery curve quantifies GFP–RdLyz‐I1 droplet fluorescence replenishment over 180 s. Data are shown as the mean ± SEM of three droplets. E) Left: domain structure and disorder plot (dSCOPE scores) for full‐length RdLyz‐I1. Right: disorder analysis specifically for the RdLyz‐I1 C‐terminal region (L2, aa 144–179). F) Confocal microscopy reveals the subcellular localization of singly expressed GFP–RdLyz‐I1, GFP–RdLyz‐I1^△L2^, and mCherry–RGDV Pns9 in HEK‐293T cells. Coverslips were collected at 48 h post‐transfection (hpt) for imaging. Scale bars: 5 µm. G) Quantification of the mean number of RdLyz‐I1 condensates per HEK‐293T cell expressing GFP–RdLyz‐I1 or GFP–RdLyz‐I1^△L2^. Data shown are representative of three biological replicates. H) Confocal microscopy reveals subcellular distribution of mCherry–RGDV Pns9 when coexpressed with GFP–RdLyz‐I1 or GFP–RdLyz‐I1^△L2^ in HEK‐293T cells. Coverslips were collected at 48 hpt for imaging. Scale bars: 5 µm. I) Western blot analysis of RGDV Pns9 cleavage and RdLyz‐I1 expression in HEK‐293T cells cotransfected for 24 h with 1.0 µg Flag–RGDV Pns9–Myc plasmids alongside 2.0 µg of either HA‐RdLyz‐I1 or HA‐RdLyz‐I1^△L2^ expression plasmids. Proteins were detected using anti‐Flag and anti‐HA antibodies. (G) Data are presented as mean ± SEM. **, *p* < 0.01 (Student’ s *t*‐test). (I) β‐Tubulin bands indicate equal protein loading. All data represent three biological replicates.

Liquid–liquid phase separation (LLPS) has emerged as a fundamental biophysical mechanism driving the formation of biomolecular condensates that play critical roles in viral replication and host antiviral defense.^[^ ^30^ ^]^ However, whether insect antiviral proteins directly target viral proteins through phase separation mechanisms remains unexplored. We detected RdLyz‐I1 forming condensates that colocalized with Pns9 within viruliferous leafhopper midgut cells (Figure 1G). To investigate whether RdLyz‐I1 forms these biomolecular condensates via liquid–liquid phase separation, we conducted in vitro experiments assessing purified RdLyz‐I1 phase separation. Recombinant GFP–RdLyz‐I1 protein purified from *E. coli* condensed into distinct spherical droplets within phase‐separation buffer (**Figure** **5**A). Notably, exposure to 5% 1,6‐hexanediol (HD), an established LLPS inhibitor, effectively dissolved these GFP–RdLyz‐I1 droplets (Figure 5B). Fluorescence recovery after photobleaching (FRAP) analysis revealed that GFP‐RdLyz‐I1 droplets gradually recovered fluorescence within 3 min postbleaching (Figure 5C,D), further substantiating the liquid‐phase dynamics of RdLyz‐I1 condensates. Collectively, these results confirm that RdLyz‐I1 protein autonomously undergoes phase separation to form condensates in vitro.

Given the common implication of intrinsically disordered regions (IDRs) in phase separation processes,^[^ ^31^ ^]^ we analyzed RdLyz‐I1's primary structure. This analysis predicted that residues 144–179 aa (designated L2) constitute a putative IDR, exhibiting a disorder score exceeding 0.5 (Figure 5E). Confocal microscopy confirmed that GFP–RdLyz‐I1 alone formed dynamic condensates within the cytoplasm of HEK‐293T cells (Figure 5F). Crucially, deletion of the L2 region (RdLyz‐I1^△L2^) abolished condensate formation (Figure 5F,G). Furthermore, recombinant GFP–RdLyz‐I1^△L2^ proteins failed to generate spherical condensates in vitro (Figure 5A). These findings unequivocally demonstrate that RdLyz‐I1 condensate formation critically depends on its C‐terminal IDR.

To elucidate whether RdLyz‐I1 condensates facilitate RGDV Pns9 cleavage, we analyzed the dynamics of RGDV Pns9 alongside RdLyz‐I1 and RdLyz‐I1^△L2^ in HEK‐293T cells. When expressed individually, RGDV Pns9 formed heterogeneous condensates (Figure 5F). Strikingly, coexpression of RdLyz‐I1 with RGDV Pns9 prompted the two components to merge into unified condensates (Figure 5H). Conversely, cells expressing RdLyz‐I1^△L2^ failed to form condensates, though RdLyz‐I1^△L2^ colocalized with RGDV Pns9 dispersed throughout the cytoplasm (Figure 5H). Critically, RdLyz‐I1^△L2^‐expressing cells exhibited markedly diminished RGDV Pns9 cleavage compared to RdLyz‐I1‐expressing cells (Figure 5I). These results demonstrate that RdLyz‐I1‐driven biomolecular condensates serve as functional platforms, colocalizing RGDV Pns9 and thereby enhancing the efficiency of RGDV Pns9 cleavage.

### 2.6 Leafhopper Toll Immune Signaling Pathway Induces the Expression of RdLyz‐I1

**Figure 6:**
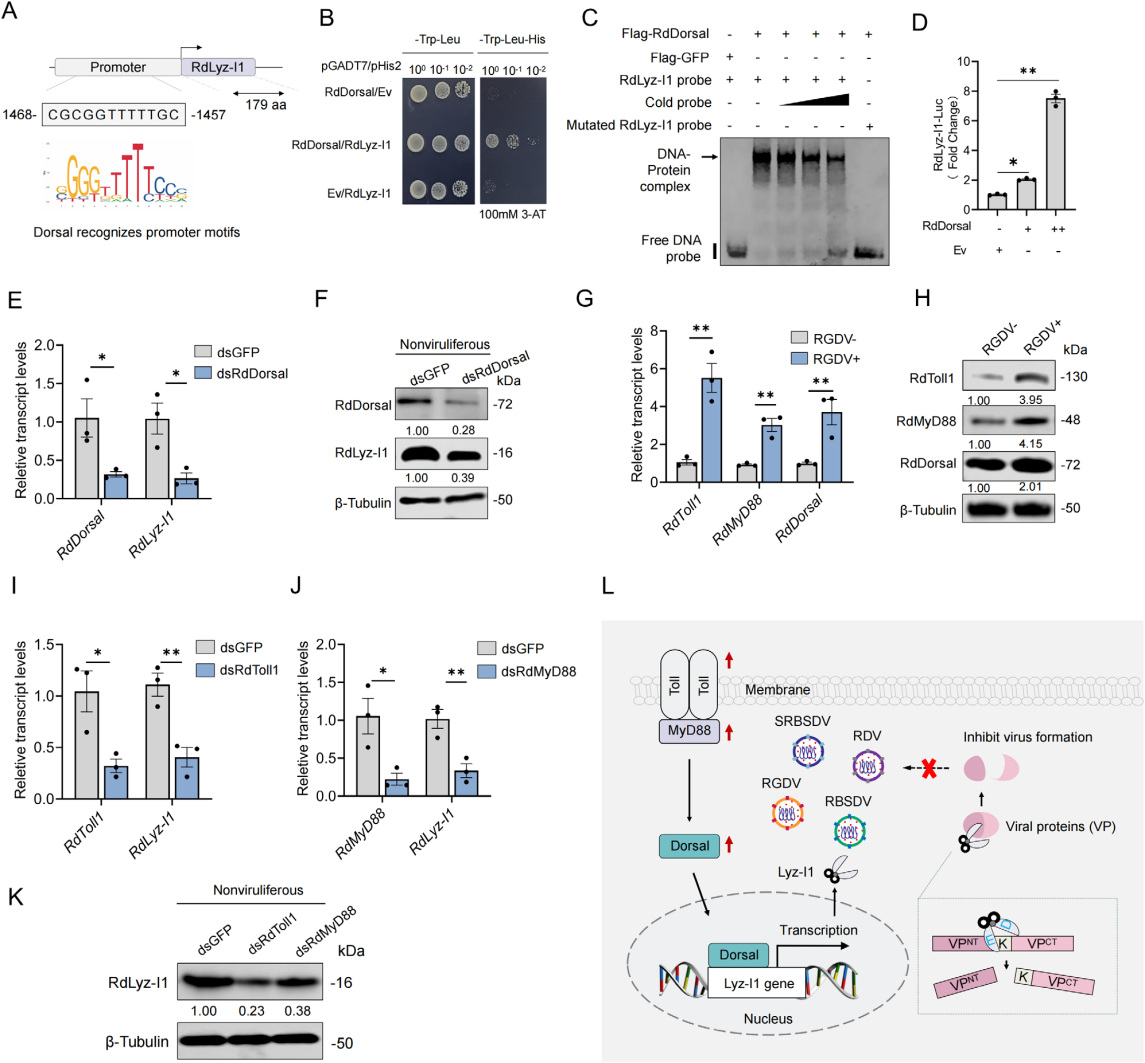
*R. dorsalis* Toll immune signaling pathway induces RdLyz‐I1 expression. A) Schematic representation of Dorsal transcription factor binding to the RdLyz‐I1 promoter region. The diagram illustrates the specific binding site located between positions −1457 to −1468 bp upstream of the *RdLyz‐I1* transcriptional start site. B) Verification of RdDorsal binding to *RdLyz‐I1* promoter sequences using Y1H assay. Yeast cells transformed with different construct combinations were cultured on DDO (SD/‐Leu/‐Trp) medium, with interactions detected on TDO (SD/‐Leu/‐Trp/‐His) media supplemented with 100 mm 3‐AT. Data represent one of three independent experiments. C) EMSA showing binding between 20 µg RdDorsal protein and 20 nmol *RdLyz‐I1* promoter probe. Different amounts of *RdLyz‐I1* promoter cold probe (0, 10, 20, and 50 nmol) competitively bound RdDorsal. GFP‐purified protein or *RdLyz‐I1* promoter mutant probe served as negative control. D) Dual‐luciferase reporter assay revealing RdDorsal‐mediated regulation of the *RdLyz‐I1* promoter in HEK‐293T cells. Cells were cotransfected with *RdLyz‐I1* promoter‐driven firefly luciferase reporter plasmids (1.0 µg), a constitutive *Renilla* luciferase control plasmid (1.0 µg), and increasing doses of Dorsal expression vector (0, 0.5 or 1.0 µg; pcDNA3.1 empty vector as control). Firefly luciferase activity was measured 48 h post‐transfection and normalized to *Renilla* luciferase activity. E,F) Effects of knocking down *RdDorsal* expression on *RdLyz‐I1* expression levels in 30 nonviruliferous leafhoppers based on RT‐qPCR (E) and western blot (F) assays. RNA and protein were extracted from 30 pooled leafhoppers per replicate. (F) Relative band intensities for RdDorsal and RdLyz‐I1 levels. G,H) RT‐qPCR (G) and western blot (H) assays showing the expression of RdToll1, RdMyD88, and RdDorsal in nonviruliferous or viruliferous leafhoppers. RNA and protein were extracted from 30 pooled leafhoppers per replicate. (H) Relative band intensities for RdToll1, RdMyD88, and RdDorsal levels. I–K) Effects of knocking down *RdToll1* and *RdMyD88* expression on *RdLyz‐I1* expression levels in nonviruliferous leafhoppers based on RT‐qPCR (I,J) and western blot (K) assays. RNA and protein were extracted from 30 pooled leafhoppers per replicate. Relative band intensities for RdLyz‐I1 levels are shown in (K). L) Proposed model of protease Lyz‐I1 cleave rice viral protein to inhibit infection. Upon rice viral infection, the Toll–MyD88–Dorsal signaling cascade is activated in insect vectors, inducing Lyz‐I1 expression. The Lyz‐I1 protein functions as a specialized antiviral protease through its conserved catalytic dyad (E/D), which mediates precise cleavage of viral proteins at specific lysine (K) residue. This site‐specific proteolytic activity disrupts essential viral protein functions, thereby effectively inhibiting viral replication and infection. (D,E,G,I,J) Data are presented as mean ± SEM. *, *p* < 0.05; **, *p* < 0.01 (Student’ s *t*‐test). (F,H,K) β‐Tubulin bands indicate equal protein loading. All data represent three biological replicates.

Dorsal functions as a pivotal transcription factor within the Toll signaling pathway, and its nuclear translocation is essential for activating downstream AMP genes.^[^ ^32^ ^]^ To comprehensively understand this antiviral pathway, we sought to determine whether the leafhopper RdDorsal regulates *RdLyz‐I1* expression. We obtained the 2000 bp putative promoter sequences of *RdLyz‐I1* from *R. dorsalis* through genomic analysis. By utilizing the JASPAR website (https://jaspar.elixir.no/), we identified a Dorsal binding site within the *RdLyz‐I1* promoter region (**Figure** **6**A).

We employed the yeast one‐hybrid (Y1H) system to determine whether RdDorsal regulates *RdLyz‐I1*. As expected, RdDorsal bound directly to the *RdLyz‐I1* promoter (Figure 6B). Electrophoresis mobility shift assay (EMSA) results demonstrated that the RdDorsal protein retarded the shift speed of the Cy5‐labeled *RdLyz‐I1* promoter probes, and this shift was competed by unlabeled probes but remained unaffected by Cy5‐labeled mutated probes, confirming the specific binding interaction between RdDorsal and the *RdLyz‐I1* promoter (Figure 6C). Furthermore, a transient transcriptional activity assay using a luciferase reporter revealed that RdDorsal directly activated *RdLyz‐I1* transcription (Figure 6D). Finally, knockdown of *RdDorsal* expression via microinjection of dsRNA targeting *RdDorsal* (dsRdDorsal) into nonviruliferous leafhoppers significantly decreased *RdLyz‐I1* expression (Figure 6E,F). These results collectively indicated that RdLyz‐I1 functions downstream of and is regulated by the transcription factor RdDorsal.

We further analyzed the effect of RGDV infection on key genes expression within the leafhopper Toll pathway. RT‐qPCR and western blot assays showed that RGDV infection significantly elevated both transcript and protein accumulation levels of RdToll1, RdMyD88, and RdDorsal (Figure 6G,H). Moreover, knockdown of either *RdToll1* or *RdMyD88* expression in nonviruliferous leafhoppers significantly decreased *RdLyz‐I1* transcript and protein levels (Figure 6I–K). These findings indicate that rice virus infection activates the Toll1–MyD88–Dorsal signaling cascade, inducing antiviral RdLyz‐I1 expression. RdLyz‐I1 functions as a specialized antiviral protease via its conserved catalytic dyad E/D, mediating precise cleavage of viral proteins at specific K residue, thereby inhibiting viral replication and infection (Figure 6L).

### 2.7 RdLyz‐I1 Effectively Inhibits RGDV Infection in Rice

**Figure 7:**
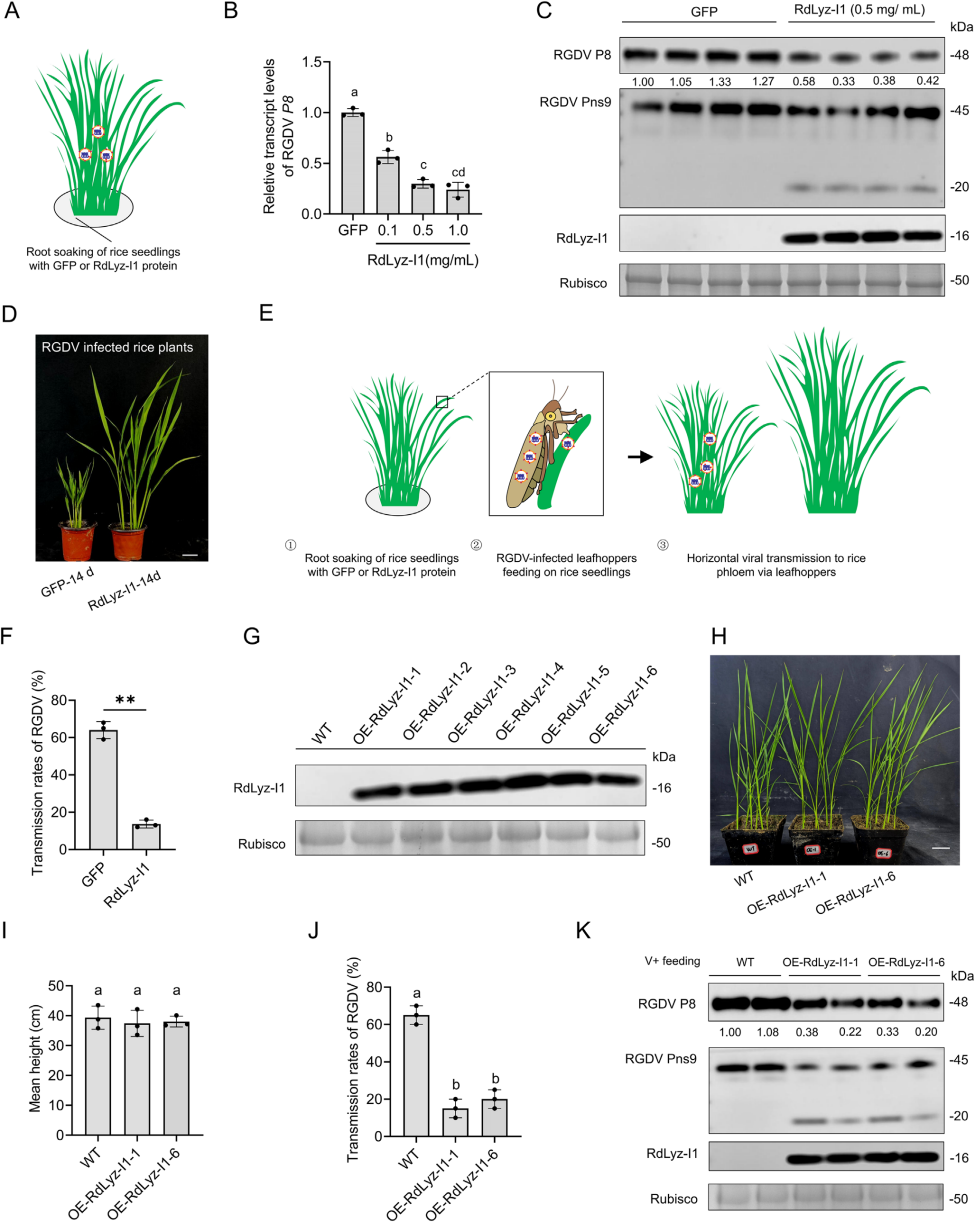
RdLyz‐I1 treatment inhibits RGDV infection in rice. A) Schematic of the experimental setup: under laboratory conditions, virus‐infected rice plants cultivated to ≈20 cm height after viruliferous leafhopper‐mediated infection were selected to hydroponically cultivate. Purified RdLyz‐I1 was added to the culture medium at different concentration of 0.1, 0.5, and 1.0 mg mL^−1^, with the medium replaced every two days. Purified GFP protein (0.5 mg mL^−1^) served as the control group. B) RT‐qPCR analysis of RGDV *P8* gene expression in virus‐infected rice treated with 0.5 mg mL^−1^ GFP (control) or different concentrations (0.1, 0.5, and 1.0 mg mL^−1^) of RdLyz‐I1 protein. Total RNA was extracted from 0.5 g leaf samples per biological replicate at 14 days postinitial protein treatment. C) Western blot analysis assessed viral proteins and RdLyz‐I1 accumulation in virus‐infected rice exposed to 0.5 mg mL^−1^ GFP and RdLyz‐I1 proteins. Protein was extracted from 0.5 g leaf samples per biological replicate at 14 days postinitial protein exposure. Relative band intensities for RGDV P8 levels are shown below. D) Phenotypic comparison of virus‐infected rice plants treated with 0.5 mg mL^−1^ GFP or RdLyz‐I1 proteins for 14 days. Scale bars: 5 cm. E) Schematic diagram of the experimental steps. First, rice seedlings ≈10 cm tall were hydroponically cultivated in culture medium containing 0.5 mg mL^−1^ RdLyz‐I1 or GFP‐purified proteins for 7 days, with the medium replaced every two days. Subsequently, five viruliferous leafhoppers were confined with one seedling using glass tubes. Seedlings were hydroponically maintained in normal culture medium during the 3‐days leafhopper feeding period before leafhopper removal. Normal cultivation continued for 10 days; leaves were then collected for RGDV detection. F) Transmission rates of RGDV analysis by viruliferous leafhopper. RT‐PCR detection of the RGDV *P8* gene in rice leaves prepared as per steps shown in (E), amplified bands indicated successful virus transmission. Twenty‐five samples per group were analyzed across three biological replicates. Data represent mean ± SEM (**, *p* < 0.01; Student's *t*‐test). G) The protein expression levels of RdLyz‐I1 in different transgenic rice lines were tested by western blot assay. H,I) The growth status (H) and the mean plant height (I) of two RdLyz‐I1‐His‐expressed transgenic lines (OERdLyz‐I1‐1 and OERdLyz‐I1‐6) as compared with the wild‐type rice plant. J) Transmission rates of RGDV analysis by viruliferous leafhopper. RT‐PCR detection of the RGDV *P8* gene in rice leaves. Twenty‐five samples per group were analyzed across three biological replicates. K) Western blot analysis assessed viral proteins accumulation in virus‐infected transgenic lines as compared with the wild‐type rice plant. Protein was extracted from 0.5 g leaf samples per biological replicate. Relative band intensities for RGDV P8 levels are shown below. (B,I,J) Data are mean ± SEM. (Different lowercase letters above the bars indicate statistically significant differences among groups as determined by one‐way ANOVA followed by Tukey's multiple comparisons test (*p* < 0.05).) (C,G,K) Rubisco large subunit served as loading control.

To evaluate the antiviral potential of exogenously applied RdLyz‐I1, purified recombinant RdLyz‐I1 protein was supplemented into rice culture medium at final concentrations of 0.1, 0.5, or 1.0 mg mL^−1^, with GFP used as a negative control. RGDV‐infected rice plants (14 days after viruliferous leafhopper transmission) were subjected to root soaking in the RdLyz‐I1‐containing medium. Leaf tissues were collected following 14 days of continuous treatment (**Figure** **7**A). RT‐qPCR analysis revealed that treatment with 0.5 mg mL^−1^ RdLyz‐I1 resulted in a more significant reduction in the transcription levels of RGDV *P8* gene compared to the 0.1 mg mL^−1^ treatment (Figure 7B). Western blot assays further confirmed that application of 0.5 mg mL^−1^ RdLyz‐I1 effectively induced proteolytic cleavage of RGDV Pns9 protein, thereby suppressing the accumulation of RGDV P8 in infected leaves (Figure 7C). These results suggest that RdLyz‐I1 was successfully absorbed through the roots and translocated to the leaves. Phenotypic assessment showed a statistically significant increase in plant height in RdLyz‐I1‐treated plants compared to virus‐infected controls (Figure 7D), demonstrating alleviation of RGDV‐induced growth retardation.

The rice fields in Yunxiao, Fujian Province, China, are located in an epidemic area for RGDV.^[^ ^33^ ^]^ We applied 0.5 mg mL^−1^ RdLyz‐I1 to rice seedlings via root soaking and subsequently cultivated the treated plants in field plots located in this RGDV‐prevalent region. Forty days after treatment, the incidence of RGDV infection in RdLyz‐I1‐treated plots was ≈12%, compared to ≈52% in phosphate‐buffered saline (PBS) control plots (Figure S4A,B, Supporting Information). These results demonstrate that RdLyz‐I1 significantly enhances antiviral resistance under realistic agricultural conditions.

We next investigated whether RdLyz‐I1 affected RGDV transmission rates by insect vectors (Figure 7E). Rice seedlings were treated with 0.5 mg mL^−1^ RdLyz‐I1 via root soaking. At 7 days post‐treatment, viruliferous leafhoppers were allowed a 3‐days acquisition access period on treated plants. Ten days after insect removal, RGDV transmission efficiency was quantified by RT‐PCR detection of the *P8* gene in rice tissues. Results showed virus transmission rate was ≈60% in the control group, but significantly reduced to ≈15% in the RdLyz‐I1‐treatment plants (Figure 7F), demonstrating that RdLyz‐I1 severely compromises vector‐mediated viral transmission.

To evaluate the sustained antiviral activity of RdLyz‐I1, we generated transgenic rice plants that constitutively overexpressed RdLyz‐I1 (Figure 7G). The RdLyz‐I1‐expressing transgenic rice lines exhibited a phenotypic profile similar to that of wild‐type controls (Figure 7H,I). Transgenic rice lines (OE‐RdLyz‐I1‐1 and OE‐RdLyz‐I1‐6) were then fed on viruliferous leafhoppers. Ten days after insect removal, the transmission efficiency of RGDV was quantified by RT‐PCR detection of *P8* gene in rice tissues, revealing a significant reduction in the rate of viral transmission by viruliferous leafhoppers compared to wild‐type rice plants (Figure 7J). Western blot analysis further demonstrated that RdLyz‐I1 mediated proteolytic cleavage of RGDV Pns9, leading to suppressed accumulation of P8 in infected leaves (Figure 7K), consistent with those obtained through exogenous application of RdLyz‐I1.

To combat pathogen invasion, plants have evolved a sophisticated two‐layer immune system, comprising effector‐triggered immunity (ETI) and PAMP‐triggered immunity (PTI).^[^ ^34^ ^]^ Within this system, intracellular nucleotide‐binding leucine‐rich repeat receptors (NLRs) and pattern‐recognition receptor (PRRs) recognize pathogenic threats, initiating potent ETI and PTI signaling cascades.^[^ ^34^ ^]^ Concurrently, key phytohormones including salicylic acid (SA) and jasmonic acid (JA) orchestrate defense responses against diverse pathogens. Given emerging evidence that small molecules can prime plant immunity,^[^ ^35^ ^]^ we investigated whether exogenous application of RdLyz‐I1, beyond directly inhibiting viral proteins, also modulates immune gene expression in rice. We quantified via RT‐qPCR the expression of key immune regulators in rice leaves seven days after RdLyz‐I1 treatment, including five NLR genes (*PigmR*, *SUMM2*, *RPM1*, *P1C1*, and *Bph14*), one PRR gene (*FLS2*), three SA synthesis‐related genes (*NPR1*, *LOX1*, and *PAL1*), and two JA synthesis‐related genes (AOS1 and EDS5). Notably, NLR genes *SUMM2*, *RPM1*, *Bph14*, PRR gene *FLS2*, and SA synthesis‐related genes *NPR1*, *LOX1*, *PAL1* were significantly upregulated (Figure S5, Supporting Information). These results demonstrate that RdLyz‐I1 also activates rice immune defenses through coordinated induction of ETI, PTI, and SA signaling pathways, suggesting an indirect mechanism that contributes to viral resistance.

## 3 Discussion

This study redefines our understanding of lysozymes function by revealing a novel enzymatic antiviral mechanism that repurposes the enzyme's conserved catalytic machinery. For nearly a century since Fleming's seminal discovery, lysozymes have been characterized as hydrolytic enzymes targeting bacterial peptidoglycan through cleavage of β‐1,4‐glycosidic bonds between NAM and NAG, mediated by a conserved Glu/Asp catalytic dyad.^[^ ^1^, ^2^, ^4^ ^]^ Our findings reveal that insect i‐type lysozymes have repurposed their ancient catalytic core for site‐specific viral protein cleavage. This discovery transcends the nonenzymatic antiviral roles occasionally attributed to lysozymes, instead establishing them as sequence‐specific antiviral proteases.

The molecular mechanism underlying this novel antiviral activity centers on the catalytic dyad E34/D50 of RdLyz‐I1 recognizing and cleaving at a specific lysine residue (K180) within the S2 domain of RGDV Pns9. While maintaining the characteristic five α‐helix fold and catalytic dyad E34/D50 that is structurally homologous to the Glu35/Asp52 dyad of hen egg‐white lysozyme, RdLyz‐I1 has remarkably adapted this conserved framework for hydrolyzing peptide bonds rather than its ancestral glycosidic linkages. This represents a functional divergence from canonical lysozyme activity, where the enzyme's target shifts from the repetitive polysaccharide structure of bacterial peptidoglycan to specific amino acid residues within viral proteins. The bond cleavage specificity undergoes a fundamental transformation from glycosidic (C─O─C) to peptide (C─N), with RdLyz‐I1 targeting the bond adjacent to the amino group of K180. The optimal conditions for this proteolytic activity (pH 5.0, 20 °C) show potential differences from those typically associated with peptidoglycan hydrolysis,^[^ ^36^ ^]^ suggesting evolutionary adaptation of the active site microenvironment while maintaining the core catalytic residues. Notably, although the acquisition of new enzymatic functions often involves evolutionary trade‐offs that reduce native activity,^[^ ^37^ ^]^ RdLyz‐I1 retains detectable bacterial peptidoglycan‐degrading activity. This illustrates the dual functionality of its catalytic site, which can accommodate structurally diverse substrates, potentially through subtle conformational adjustments or differential utilization of adjacent residues.

The discovery of this proteolytic activity places lysozymes within the broader framework of host–virus interaction. Current research has characterized how viral proteases target and cleave host immune proteins to subvert antiviral defenses. For example, flavivirus proteases disrupt the cGAS–STING pathway by cleaving human cGAS and STING,^[^ ^38^ ^]^ while picornavirus 3C proteases directly cleave key adaptors MAVS and TRIF to inhibit RLR/TLR‐mediated interferon production.^[^ ^39^ ^]^ In striking contrast, our knowledge of host‐encoded proteases that directly target and cleave viral proteins to restrict infection remains notably limited.^[^ ^40^ ^]^ Our findings reveal an evolutionary counter strategy where host lysozymes have been repurposed as antiviral proteases. This discovery significantly expands our understanding of host proteases that directly cleave viral proteins to restrict infection and highlights the crucial importance of the lysozyme family in immune defense mechanisms.

The antiviral efficacy of RdLyz‐I1 is significantly enhanced through LLPS, a mechanism that enhances enzymatic activity through spatial organization. Structural analysis reveals that a C‐terminal IDR drives the formation of dynamic biomolecular condensates by RdLyz‐I1, which selectively recruits Pns9 viral substrates. This co‐condensation creates a specialized catalytic microenvironment that significantly enhances proteolytic efficiency beyond simple substrate colocalization. The phase‐separated condensates function as specialized reaction crucibles, ensuring maximal antiviral impact precisely at viral replication sites where RdLyz‐I1 and Pns9 extensively colocalize, potentially through a combination of increased local concentration and optimized reaction conditions. This mechanism parallels observations in mammalian systems where interferon‐induced GTPase MX2 forms biomolecular condensates that act as nuclear pore decoys, capturing the HIV‐1 capsid and inhibiting viral replication.^[^ ^31^ ^]^ Collectively, these observations establish biomolecular condensate formation as an evolutionarily conserved biophysical strategy in host–pathogen interaction, crucially regulating enzymatic immune responses.

This antiviral mechanism, which involves Lyz‐I1‐mediated proteolysis of viral replication proteins, inhibits the propagation of plant reoviruses in insect vectors and demonstrates significant evolutionary conservation across various insect vector‐reovirus systems. Comparative analysis reveals that orthologous i‐type lysozymes in other major rice virus vectors, including *N. cincticeps* (RDV vector), *S. furcifera* (SRBSDV vector), and *L. striatellus* (RBSDV vector), preserve both the characteristic structural architecture and catalytic dyad residues. Importantly, these lysozyme homologs exhibit conserved targeting specificity toward viral replication proteins, indicating a shared molecular recognition paradigm. The invariant dependence on catalytic dyads and the consistent selection of lysine residues across viral targets strongly supports the existence of a conserved antiviral strategy that is widely conserved within the innate immune systems of these insect vectors, representing an evolutionary adaptation to persistent selection pressures exerted by related plant reoviruses. This mechanism likely represents an ancient insect antiviral strategy that balances viral propagation and vector survival by selectively cleaving viral replication proteins, thereby ensuring persistent viral transmission and vector coexistence.

Our findings establish that the canonical insect Toll pathway regulates this enzymatic antiviral mechanism through Dorsal‐mediated transcription. This represents a significant expansion of Toll pathway effectors beyond classical AMP mechanisms involving membrane disruption or peptidoglycan hydrolysis,^[^ ^13^, ^15^ ^]^ instead demonstrating a novel effector function characterized by precise enzymatic targeting of viral proteins. These findings indicate that AMPs can also function as specific proteolytic scissors, disabling viruses by cleaving essential viral components crucial for replication. The Toll–Dorsal–Lyz‐I1 axis exhibits remarkable evolutionary conservation, paralleling observations in invertebrates like shrimp (*Litopenaeus vannamei*), where Dorsal similarly regulates lysozyme expression during antiviral responses.^[^ ^18^ ^]^ Given the universal conservation of NF‐κB homologs and i‐type lysozymes across insects, Toll–Dorsal mediated Lyz‐I1 induction likely represents a phylogenetically conserved and widespread antiviral strategy.

Exogenous antiviral agents can reduce viral titers and alleviate plant disease symptoms, often by activating host defense pathways.^[^ ^35^ ^]^ We demonstrate efficient control of RGDV in rice through root soak delivery of RdLyz‐I1, which translocates to leaf tissues and cleaves RGDV Pns9, triggering its degradation. This treatment reduces viral accumulation, and activates plant immune defenses. Collectively, our work identifies a previously unrecognized function for lysozymes: the repurposing of their conserved catalytic machinery for the site‐specific cleavage of viral proteins, representing a targeted antiviral strategy. This paradigm shift expands our understanding of lysozyme biology and innate immune defense mechanisms while establishing foundation for novel crop protection strategies.

## 4 Experimental Section

### Plants, Insects, Cells, and Antibodies

Nonviruliferous *R. dorsalis* populations were maintained in a greenhouse under constant conditions: 25 ± 1 °C, 75% ± 5% relative humidity, and a 16 h light/8 h dark photoperiod. The RGDV‐infected rice (*Oryza sativa*) plants were originally collected from Luoding City, Guangdong Province, China. They were propagated through successive insect‐mediated transmission cycles. Rice seedlings were hydroponically cultivated using Murashige and Skoog (MS) medium (Phytotech, Cat# M519) under precisely controlled conditions mirroring the *R. dorsalis* rearing environment.

HEK‐293T human embryonic kidney cells were maintained in Dulbecco's Modified Eagle Medium (Gibco, Cat# 10566016) containing 10% FBS (Gibco, Cat#10099141C) at 37 °C in a humidified 5% CO_2_ incubator.

Custom rabbit polyclonal antibodies against *R. dorsalis* RdLyz‐I1, RdToll1, RdMyD88, RdDorsal, and RGDV P8, RGDV Pns9 generated through antigen peptide immunization as previously described.^[^ ^41^ ^]^ Commercial antibodies included: β‐Tubulin (TransGen Biotech, HC101), Myc Tag (TransGen Biotech, HT101), Flag Tag (TransGen Biotech, HT201), HA Tag (TransGen Biotech, HT301), His Tag (TransGen Biotech, HT501) and GST Tag (TransGen Biotech, HT601), histone H3 (TransGen Biotech, HL1021), and HRP‐conjugated secondary antibodies (goat anti‐rabbit IgG, Thermo Fisher 32460; goat anti‐mouse IgG, Thermo Fisher 32430).

### RT‐qPCR Analysis

To quantify gene expression levels of *RdToll1*, *RdMyD88*, *RdDorsal*, *RdLyz‐I1*, *RdLyz‐I2*, *RdLyz‐C1*, and *RdLyz‐C2* in *R. dorsalis*, as well as the effect of RdLyz‐I1 treatment on rice immune genes, total RNA from 50 intact insects infected with RGDV (6 days padp) or treated with dsRNAs as well as 100 mg rice leaves treated with RdLyz‐I1 or GFP control was extracted using TRIzol reagent following the manufacturer's instructions. First‐strand cDNA synthesis was performed with 1 µg total RNA using RevertAid Reverse Transcriptase (Promega, EP0441) and oligo(dT) primers. RT‐qPCR reactions were conducted in triplicate using the SYBR Green PCR Master Mix (GenStar, A304‐10) on a QuantStudio 6 Flex Real‐Time PCR System (Applied Biosystems). Relative gene expression was calculated using the 2^−ΔΔCt^ method, with *R. dorsalis elongation factor 1* (*EF1*) or *O. sativa Actin* as endogenous controls. Primer sequences are provided in Table S1 of the Supporting Information.

### Western Blot Analysis

Total proteins were extracted from 50 nonviruliferous, viruliferous, or dsRNAs‐treated intact insects or 100 mg rice leaves treated with RdLyz‐I1 or GFP control using RIPA lysis buffer (Beyotime, P0013B) containing protease inhibitor cocktail (Roche, 11697498001). Proteins were transferred to PVDF membranes (Millipore, IPFL00010), blocked with 5% nonfat milk, and probed with primary antibodies (1:1000 dilution) overnight at 4 °C. After incubation with HRP‐conjugated secondary antibodies (1:5000), signals were developed using Luminata Classico Western HRP Substrate (Millipore, WBKLS0500) and imaged on a Luminescent Image Analyzer AI600 (GE Healthcare). Band intensities were quantified using ImageJ software (NIH).

### Immunofluorescence Microscopy

Midgut tissues from RGDV‐free or RGDV‐infected adults (6 days padp) were fixed in 4% paraformaldehyde, permeabilized with 0.2% Triton X‐100, and incubated with rhodamine‐conjugated RdLyz‐I1 and FITC‐conjugated RGDV Pns9 antibodies (0.5 µg mL^−1^) overnight at 4 °C. Images were acquired on a Leica TCS SPE confocal microscope using a 100× oil immersion objective.

### Transmission Electron Microscopy (TEM)

Midgut tissues from RGDV‐free or RGDV‐infected adults (6 days padp) were dissected and fixed in 2% (v/v) glutaraldehyde and 2% (v/v) paraformaldehyde in 0.1 m PBS (pH 7.4) for 2 h at room temperature, followed by postfixation in 1% (w/v) osmium tetroxide for 1 h. Samples were dehydrated through an ethanol series (30–100%), embedded in Spurr's resin (SPI Supplies, 02680‐AB), and polymerized at 60 °C for 72 h. Ultrathin sections (70 nm) were cut using a Leica EM UC7 ultramicrotome, mounted on nickel grids, and immunolabeled with RdLyz‐I1‐specific IgG or RGDV Pns9‐specific IgG (0.5 µg mL^−1^) followed by 10 nm gold‐conjugated goat anti‐rabbit secondary antibody (Abcam, ab27236). Grids were counterstained with uranyl acetate and lead citrate before imaging on a Hitachi HT‐7800 TEM operated at 80 kV.

### Yeast Two‐Hybrid (Y2H) Assay

Y2H assays were employed to investigate protein–protein interactions among RdLyz‐I1 and its homologs with viral proteins. Open reading frames (ORFs) of *RdLyz‐I1*, *RdLyz‐I2*, *RdLyz‐C1*, *RdLyz‐C2, SfLyz‐I1*, *LsLyz‐I1*, *NcLyz‐I1* (without the signal peptide) were cloned into the bait vector pGBKT7. For prey vector construction, full‐length ORFs of RGDV proteins (*P3*, *P4*, *P5*, *P6*, *Pns7*, *P8*, *Pns9*, *P10*, *Pns11*, and *Pns12*), SRBSDV *Pns9*, RBSDV *Pns9*, RDV *P7*, along with RGDV *Pns9* segments deletion mutants Pns9^△S1^, Pns9^△S2^, and Pns9^△S3^ were into the vector pGADT7. Yeast strain AH109 was cotransformed with bait/prey plasmids using the LiAc/SS carrier DNA/PEG method and plated on SD/‐Trp–Leu–His–Ade (QDO) medium. The pGBKT7‐53/pGADT7‐T interaction served as a positive control, while the pGBKT7‐Lam/pGADT7‐T interaction served as a negative control. Positive interactions were confirmed by colony growth after 5 days at 30 °C. Primer sequences are listed in Table S1 of the Supporting Information.

### Glutathione S‐Transferase (GST) Pull‐Down Assay

GST pull‐down assays were used to confirm the interaction among RdLyz‐I1 and viral proteins. GST‐tagged RGDV Pns9 was cloned into pGEX‐4T‐3 and His‐tagged proteins (RdLyz‐I1, RdLyz‐I2, RdLyz‐C1, RdLyz‐C2) cloned into pET28a were expressed in *E. coli* Rosetta (DE3) cells. GST fusion proteins were immobilized on glutathione‐sepharose 4B beads (GE Healthcare, 17075601) and incubated with His_6_‐tagged proteins in binding buffer (50 mm Tris‐HCl pH 7.5, 150 mm NaCl, 0.1% NP‐40) for 4 h at 4 °C. Beads were washed five times with ice‐cold PBS, and bound proteins were eluted with 10 mm reduced glutathione. Interactions were verified by western blot using anti‐His (1:3000) and anti‐GST (1:2000) antibodies. Primer sequences are listed in Table S1 of the Supporting Information.

### Protein Expression and Purification

The coding sequences for RdLyz‐I1 (without the signal peptide) and RGDV Pns9, along with relevant mutants, were cloned into the pET‐28a vector, generating N‐terminal His_6_‐tagged fusion constructs. Recombinant plasmids were transformed into *E. coli* BL21 (DE3) competent cells and selected on LB agar plates containing kanamycin (50 µg mL^−1^). For large‐scale expression, a single colony was inoculated into 50 mL of LB medium with antibiotics and grown overnight at 37 °C, 220 rpm. This starter culture was then diluted 1:100 into 2 L of fresh LB medium with antibiotic and grown at 37 °C until the optical density at 600 nm (OD_600_) reached 0.6–0.8. The cultures were then cooled on ice for 20 min, induced with 1 mm isopropyl β‐d‐1‐thiogalactopyranoside (IPTG), and further incubated for 18–20 h at 18 °C, 180 rpm. The bacteria were harvested and resuspended in Lysis Buffer (50 mm Tris, pH 8.0, 500 mm NaCl, 20 mm imidazole, 10% (v/v) glycerol) supplemented with 1 mm phenylmethylsulfonyl fluoride. Bacteria lysis was performed by sonication on ice (10 min total process time, 5 s on, 10 s off). The lysate was clarified by centrifugation at 16 000 × *g* for 30 min at 4 °C, and the supernatant was filtered through a 0.45 µm membrane.

The filtered supernatant was loaded onto a 5 mL HisTrap HP column pre‐equilibrated with Lysis Buffer. The column was washed with 10 column volumes of Lysis Buffer to remove nonspecifically bound proteins. Bound protein was eluted using a linear gradient of Elution Buffer (50 mm Tris, pH 7.5, 300 mm NaCl, 500 mm imidazole, 10% glycerol). Eluted protein fractions were subsequently purified by Hiload 16/600 Superdex 200 pg column equilibrated with GF Buffer (20 mm Tris pH 7.5, 150 mm NaCl), followed by ion‐exchange chromatography on a Resource S column using Buffer A (10 mm Tris, pH 8.0, 100 mm NaCl) and Buffer B (10 mm Tris, pH 8.0, 1 m NaCl). Purified proteins were concentrated to ≈10 mg mL^−1^ using Amicon Ultra centrifugal filters (10 kDa MWCO for RdLyz‐I1; 30 kDa MWCO for Pns9), flash‐frozen in liquid nitrogen, and stored at −80 °C in storage buffer containing 5 mm DTT and 5% glycerol. Protein concentration was determined by the Bradford assay using BSA as a standard. Purity and identity were confirmed by SDS‐PAGE followed by silver staining, using the Fast Silver Stain Kit (Beyotime, P0017S).

### Isothermal Titration Calorimetry (ITC) Experiments

The binding affinity of RdLyz‐I1 to viral proteins and their mutants was quantified by ITC using a MicroCal PEAQ‐ITC system (Malvern Panalytical). Prior to measurements, all protein samples were equilibrated in degassed buffer (50 mm Tris, 300 mm NaCl, pH 7.5). Titrations were performed at 25 °C with constant stirring (750 rpm), injecting 19 aliquots (2 µL per injection) of protein solution from the syringe into the sample cell at 150 s intervals. Data were fitted to a one‐set‐of‐sites binding model. Results represent mean ± SEM from triplicate independent experiments, with ITC thermograms showing representative data.

### RNA Interference (RNAi)

The dsRNAs targeting *RdLyz‐I1, RdLyz‐I2, RdLyz‐C1*, *RdLyz‐C2*, *RdToll1*, *RdMyD88*, *RdDorsal*, and *GFP* were synthesized using the T7 RiboMAX Express RNAi system (Promega, P1700). The primers used for dsRNA synthesis are listed in Table S1 of the Supporting Information. Approximately 500 newly emerged leafhoppers were allowed to feed on RGDV‐infected rice plants for two days before feeding on healthy rice seedlings for one day to evaluate the effect of the dsRNAs on virus or indicated proteins accumulation. Nonviruliferous and viruliferous insects were independently microinjected with 46 nL of dsRNAs at 1 µg µL^−1^ for each gene (≈50 ng per adult) using a Nanoject II Auto‐Nanoliter Injector (Spring). The dsGFP treatment served as a control. Three days postinjection, transcript, and protein levels of target genes were analyzed by RT‐qPCR and western blot assays.

### Protein Microinjection Assays

The protein microinjection method has been extensively validated across diverse insect species.^[^ ^19^, ^29^, ^41^, ^42^ ^]^ Recombinant RdLyz‐I1, RdLyz‐I2, RdLyz‐C1, and RdLyz‐C2 proteins expressed in *E. coli* were purified using Ni‐NTA affinity chromatography (Qiagen, 30210). Approximately 300 viruliferous leafhopper adults at 6 days padp were microinjected with 1 µg µL^−1^ purified protein to detect their effects on RGDV accumulation. At 48 h postinjection, 50 insects per replicate were pooled, and total protein was extracted using NP40 lysis buffer and analyzed by western blot. GFP protein (1 µg µL^−1^) served as a negative control. Three biological replicates were performed.

### Protein Structure Prediction and Protein–Protein Docking

Protein structures of RdLyz‐I1, NcLyz‐I1, SfLyz‐I1, LsLyz‐I1, and RGDV Pns9 were predicted using Alphafold3 (DeepMind) and validated via SAVES v6.1 (https://saves.mbi.ucla.edu/). Molecular docking was performed using GRAMM‐X (http://vakser.compbio.ku.edu/resources/gramm/grammx/) with default parameters. The top‐scoring complexes were analyzed using PDBePISA (https://www.ebi.ac.uk/pdbe/pisa/) and visualized in ChimeraX 1.4.

### In Vitro Cleavage Assay and Mass Spectrometry

For the cleavage assay in vitro, 10 µg Flag‐RGDV Pns9–Myc recombinant protein incubated with different amounts of purified RdLyz‐I1 protein (0, 0.5, 2.5, and 12.5 µg) in a 50 µL reaction containing 50 mm Tris (PH 7.5), 150 mm NaCl, 0.005% (vol/vol) Tween‐20, and 10 mm DTT at 37 °C for 2 h. The cleavage reaction was terminated by adding SDS‐PAGE loading dye. The reaction mixtures were analyzed by SDS‐PAGE and stained with coomassie blue dye for 30 min. The major cleavage product band with ≈20 kDa was excised from the SDS‐PAGE gel and digested by trypsin protease for mass spectrometry. The mass spectrometry results were analyzed using Mascot software (Matrix Science).

### In Vitro Enzyme Activity Analysis

Purified peptidoglycan from *E. coli* (LMAI Bio, LM1179, 100 µg) was incubated with either RdLyz‐I1 (10 µg), RdLyz‐I2 (10 µg), or RdLyz‐C1 (10 µg) in 0.01 m PBS (pH 7.4). Purified GST (10 µg) and HEWL (Beyotime, ST206, 10 µg) served as negative and positive controls, respectively. Enzymatic activity was assessed by monitoring the optical density at 30 min intervals over 6 h at 25 °C using a microplate reader (BioTek Synergy H1, USA), with intermittent shaking. Reduced absorbance correlates with enhanced peptidoglycan degradation, reflecting higher amidase activity.

RdLyz‐I1 or RdLyz‐I1^ED/AA^ (10 µg) was dissolved in phosphate buffers at varying pH levels (3.0, 5.0, 7.0, and 9.0) and incubated at 25 °C for 4 h. Subsequently, the enzyme was mixed with 10 µg of purified RGDV Pns9 protein for 30 min. The cleavage products of Pns9 were analyzed by western blot to evaluate the pH‐dependent stability of RdLyz‐I1 activity.

RdLyz‐I1 or RdLyz‐I1^ED/AA^ (10 µg) was prepared in phosphate buffer at its optimal pH and subjected to different temperatures (20, 30, 40, 50, and 60 °C) for 4 h. Following incubation, the enzyme was reacted with 10 µg of purified Pns9 for 30 min, and cleavage products were analyzed by western blot to determine the temperature optimum for enzymatic activity. All experiments were performed in triplicate to assess variations in enzyme activity under different pH and temperature conditions, enabling comprehensive characterization of RdLyz‐I1's enzymatic properties and stability.

### Detection of Viral Proteins Cleavage by RdLyz‐I1 and Its Homologs in Cells

To investigate whether RGDV Pns9 is cleaved by RdLyz‐I1 in cells, HEK‐293T cells were transfected with recombinant plasmids expressing either RGDV Pns9 or its mutants (1.0 µg) alone, or cotransfected with RdLyz‐I1 (or its mutants) at 0.5, 1.0, or 2.0 µg. Transfections were performed in 6‐well plates using Lipo8000 transfection reagent (Beyotime, C0533). At 24 hpt, cells were lysed with NP‐40 buffer containing protease inhibitors and subjected to western blot analysis. Membranes were probed with anti‐Flag, anti‐Myc, and anti‐HA antibodies to detect the N‐terminus of RGDV Pns9, C‐terminus of RGDV Pns9, and RdLyz‐I1, respectively. After incubation with secondary antibodies, signals were captured using a Luminescent Image Analyzer AI600 (GE Healthcare).

Parallel cleavage assays were performed for SRBSDV Pns9, RBSDV Pns9, and RDV P7 using their cognate Lyz‐I1 homologs (SfLyz‐I1, LsLyz‐I1, and NcLyz‐I1, respectively) following identical experimental conditions. HEK‐293T cells expressing viral proteins alone (1.0 µg plasmids) or coexpressing Lyz‐I1 homologs (2.0 µg plasmids) were tested. Mutant variants of both viral proteins and Lyz‐I1 homologs were similarly evaluated. At 24 hpt, cells were harvested and lysed in NP‐40 buffer supplemented with protease inhibitor. Protein lysates were subsequently analyzed by western blot to assess viral protein cleavage events.

### In Vitro RdLyz‐I1 Condensates Formation Assays

In vitro, condensate formation was analyzed as previously described with minor modifications.^[^ ^43^ ^]^ Briefly, recombinant GFP, GFP–RdLyz‐I1 or GFP–RdLyz^△L2^ protein expressed in *E. coli* were purified using Ni‐NTA affinity chromatography (Qiagen, 30210), and then centrifuged at 15 000 × *g* for 10 min to remove aggregates. GFP, GFP–RdLyz‐I1 or GFP–Lyz^△L2^ (final concentration, 10 µm) were incubated within a buffer containing 150 mm NaCl, 30 mm Tris‐HCl (pH 7.5), and 1 mm DTT in a total reaction volume of 50 µL. Samples were incubated at room temperature for 5 min before imaging to allow condensates to settle. The phase‐separation assays for the dependency of salt concentrations and treatment with 5% 1,6‐HD were undertaken under similar conditions.

To investigate whether the relocalization of RGDV Pns9 to the structures induced by RdLyz‐I1 and its mutant in vitro, HEK‐293T cells were transfected with recombinant plasmids expressing GFP–RdLyz‐I1, GFP–RdLyz^△L2^, and mCherry–RGDV Pns9 to visualize the location of proteins. HEK‐293T cells were seeded on coverslips and then incubated with 50 µL of cell culture medium containing 1.0 µg indicated plasmids using the Lipo8000 kit (Beyotime, C0533). The coverslips were harvested at 48 hpt and then processed for immunofluorescence microscopy with a Leica TCS‐SP8 laser scanning confocal microscope.

### Prediction and Determination of RdLyz‐I1 IDRs

The IDRs were predicted with the online tool PONDR (http://www.pondr.com/) with default parameters. To investigate the IDR regions of RdLyz‐I1, the GFP–RdLyz‐I1, or GFP–RdLyz^△L2^ were individually expressed in HEK‐293T cells seeded on coverslips by 1.0 µg plasmids transfection in 24‐well cell culture plates. The coverslips were harvested at 48 hpt and then processed for immunofluorescence microscopy with a Leica TCS‐SP8 laser scanning confocal microscope.

### Fluorescence Recovery after Photobleaching

For the in vitro FRAP assay, experiments were performed with a Leica TCS‐SP8 laser scanning confocal microscope. Selected GFP–RdLyz‐I1 condensates were bleached three times with 100% laser power for 5 s using a 488 nm laser. Time‐lapse modes were used to collect recovery images over 3 min after bleaching with 10 s intervals. The recovery curves were plotted using GraphPad Prism8 software.

### Yeast One‐Hybrid (Y1H) Assay

The 5′ regulatory regions of *RdLyz‐I1* gene containing putative RdDorsal binding sites (DorBS) were cloned into pHis2 vector. ORF of the *RdDorsal* was cloned into pGADT7 vector. Yeast strain Y187 was cotransformed with pGADT7‐RdDorsal and pHis2‐RdLyz‐I1, with appropriate controls. Transformants were selected on ‐His/Leu/Trp media containing 100 mm 3‐Amino‐1,2,4‐triazole (3‐AT) at 30 °C. Images were captured 4 days after incubation.

### Electrophoretic Mobility Shift Assay (EMSA)

The ORF of the *RdDorsal* and *GFP* was individually cloned into the pcDNA 3.1 vector (pcDNA‐RdDorsal, pcDNA‐GFP). Flag–RdDorsal or Flag–GFP protein was individually expressed in HEK‐293T cells by 5.0 µg plasmids transfection for 2 days. Cell lysates immunoprecipitated using anti‐Flag magnetic beads (Beyotime, P2181S), and eluted with 3× Flag peptide. Cy5‐labeled probes (RdLyz‐I1, 5′‐ACATCGCGGTTTTTGCCGACGA‐3′) and unlabeled/mutant probes (RdLyz‐I1_mut_, ACATCACAACCCCCACCGACGA‐3′) were synthesized by Tsingke Technology. EMSA reactions containing 20 nmol probe, and 10 nmol poly(dI‐dC), 20 µg Flag–Dorsal or Flag–GFP protein were performed in EMSA buffer (10 mm Tris, pH7.5, 0.25 mm DTT, 5 mm MgCl_2_, 10 mm KCl) at 4 °C for 30 min. Competitive EMSA was performed by adding 0, 10, 20, and 50 nmol of unlabeled oligos 20 min prior to the addition of labeled oligos. Reactions were resolved on 5% non‐denaturing PAGE gels and imaged on an Odyssey CLx Imaging System (LI‐COR).

### Dual‐Luciferase Reporter Assay

The promoter regions of RdLyz‐I1 were cloned into the pGL3‐Basic vector (pGL3‐RdLyz‐I1). For the dual luciferase reporter assay, HEK‐293T cells were transfected with 1.0 µg of reporter gene plasmids (pGL3‐vector, pGL3‐RdLyz‐I1), 1.0 µg of pRL‐TK renilla luciferase plasmid as an internal control, and either 0, 0.5 or 1.0 µg of pcDNA‐Dorsal expression plasmids using the Lipo8000 kit. Firefly and Renilla luciferases activities were measured using the Dual Luciferase Reporter Assay Kit (YEASEN, 11402ES60) at 48 hpt. Each experiment was performed in triplicate.

### RdLyz‐I1 Treatment Rice Plants Inhibit Virus Infection and Transmission

To assess RdLyz‐I1‐mediated antiviral activity, preinfected rice plants (≈20 cm tall) carrying RGDV via viruliferous leafhoppers transmission were selected. These infected plants underwent RdLyz‐I1 (0, 0.1, 0.5 or 1.0 mg mL^−1^) or GFP (0.5 mg mL^−1^) treatments for 14 days. Leaves were then harvested for viral gene and protein quantification.

To evaluate RdLyz‐I1's effect on viral transmission, a separate group of ≈10 cm seedlings was first immersed in 0.5 mg mL^−1^ RdLyz‐I1‐ or GFP‐containing medium for 7 days. Following this, five viruliferous leafhoppers were confined per seedling using glass tubes during a 3‐days acquisition access period. After leafhopper removal, plants were cultured in standard medium for 10 days before RGDV detection. RT‐PCR detected the RGDV *P8* gene in rice leaves, with amplified bands confirming successful virus transmission.

To evaluate the practical control efficacy of RdLyz‐I1 under field conditions, rice plants were treated with 0.5 mg mL^−1^ purified RdLyz‐I1 protein using a root soaking method. Rice seedlings at the 3–4 leaf stage (≈10 cm in height) were first immersed in the RdLyz‐I1 protein solution for 12 h before transplanting. The seedlings were then transplanted into experimental paddy fields under natural conditions with standard agronomic practices. Three independent replicate plots were established for both the RdLyz‐I1 treatment and buffer‐only control groups, with each plot containing 30 plants. After transplanting, the protein solution (500 mL per plot) was reapplied by irrigation around the root zone every 15 days for a total of three applications (including the pretransplant soaking). Plants were naturally exposed to viruliferous leafhoppers (*R. dorsalis*) under field epidemic pressure. Leaf samples were collected from the newest fully expanded leaves of 25 randomly selected plants per plot at 40 days after initial treatment. Viral infection was assessed using RT‐PCR targeting the RGDV *P8* gene. Disease incidence was calculated as the percentage of infected plants per plot.

To evaluate the sustained antiviral activity of RdLyz‐I1, transgenic rice plants of the (ecotype *O. sativa L. spp. japonica*) were generated to express RdLyz‐I1‐His via *Agrobacterium tumefaciens* (GV3101)‐mediated transformation. Positive transgenic lines (OERdLyz‐I1) were selected on antibiotic‐containing media and confirmed by western blot analysis using an anti‐His antibody, with wild‐type plants serving as the control. To assess the influence of RdLyz‐I1 expression on viral transmission, five viruliferous *R. dorsalis* adults were caged on each OERdLyz‐I1 and wild‐type plant for a 3‐days acquisition access period. After removal of the insects, plants were maintained under standard growth conditions for an additional 10 days. The virus transmission rate was determined by RT‐PCR detection of the RGDV *P8* gene in systemic leaves. Leaf samples were subsequently collected for quantification of viral protein accumulation by western blot analysis.

To investigate RdLyz‐I1‐mediated rice immunity, ≈10 cm tall *O. sativa L. spp. japonica* seedlings received treatment with purified RdLyz‐I1 (0.5 mg mL^−1^) or GFP control protein in culture medium; the medium every 2 days was refreshed. After 14 days, total RNA was extracted from 100 mg leaves using TRIzol reagent. RT‐qPCR quantified rice immune genes expression, normalized to *OsActin*.

### Gene Identification and Phylogenetic Analyses

Previous transcriptome analysis (PRJNA385671) identified four putative *lysozyme* genes in *R. dorsalis*. They were confirmed through phylogenetic analysis and domain structure characterization. The phylogenetic framework expanded upon existing studies, incorporating lysozyme sequences from 20 insect species. Candidate homologs identified via BLAST were verified using NCBI's Conserved Domain Database to authenticate domain architectures.

Predicted insect lysozyme sequences and human α‐lactalbumin (outgroup) were aligned with MAFFT (https://mafft.cbrc.jp/alignment/software). The alignments served as input for maximum‐likelihood tree construction in IQ‐TREE (http://iqtree.cibiv.univie.ac.at) using default parameters. Final phylogenetic trees were visualized and annotated in iTOL (https://itol.embl.de).

### Statistical Data Analysis

All graphs and statistical analyses were generated using GraphPad Prism software (version 8.0.1). Data were analyzed by a two‐tailed unpaired Student's *t*‐test or one‐way ANOVA with Tukey's multiple comparisons test and are expressed as mean ± SEM. The significance level was set at *p* < 0.05. (*, *p* < 0.05; **, *p* < 0.01; ns, not significant).

## Conflict of Interest

The authors declare no conflict of interest.

## Author Contributions

T.W. conceived the research. T.W. and Y.D. designed the experiments. Y.D., Y.X., M.H., and J.Y. performed experiments with assistance from Y.L. T.W. and Y.D. wrote the manuscript with contributions from all authors.

## Supporting information



Supporting Information

## Data Availability

The data that support the findings of this study are available in the Supporting Information of this article.
