# Supplementary material for: Insect I‐Type Lysozymes Function as Antiviral Proteases by Forming Biomolecular Condensates
Source: Adv Sci (Weinh). 2025 Oct 27;13(1):e14408. doi: 10.1002/advs.202514408 (PMC12766996; doi:10.1002/advs.202514408)
Supplement: Supplementary file 1 — Supporting Information [file ADVS-13-e14408-s001.docx]

Supporting Information

Insect I-Type Lysozymes Function As Antiviral Proteases By Forming Biomolecular Condensates

Yu Du, Yuqing Xiao, Manman Hu, Jinhua Yang, You Li, Taiyun Wei*

This PDF file includes:

Figures S1 to S5

Tables S1


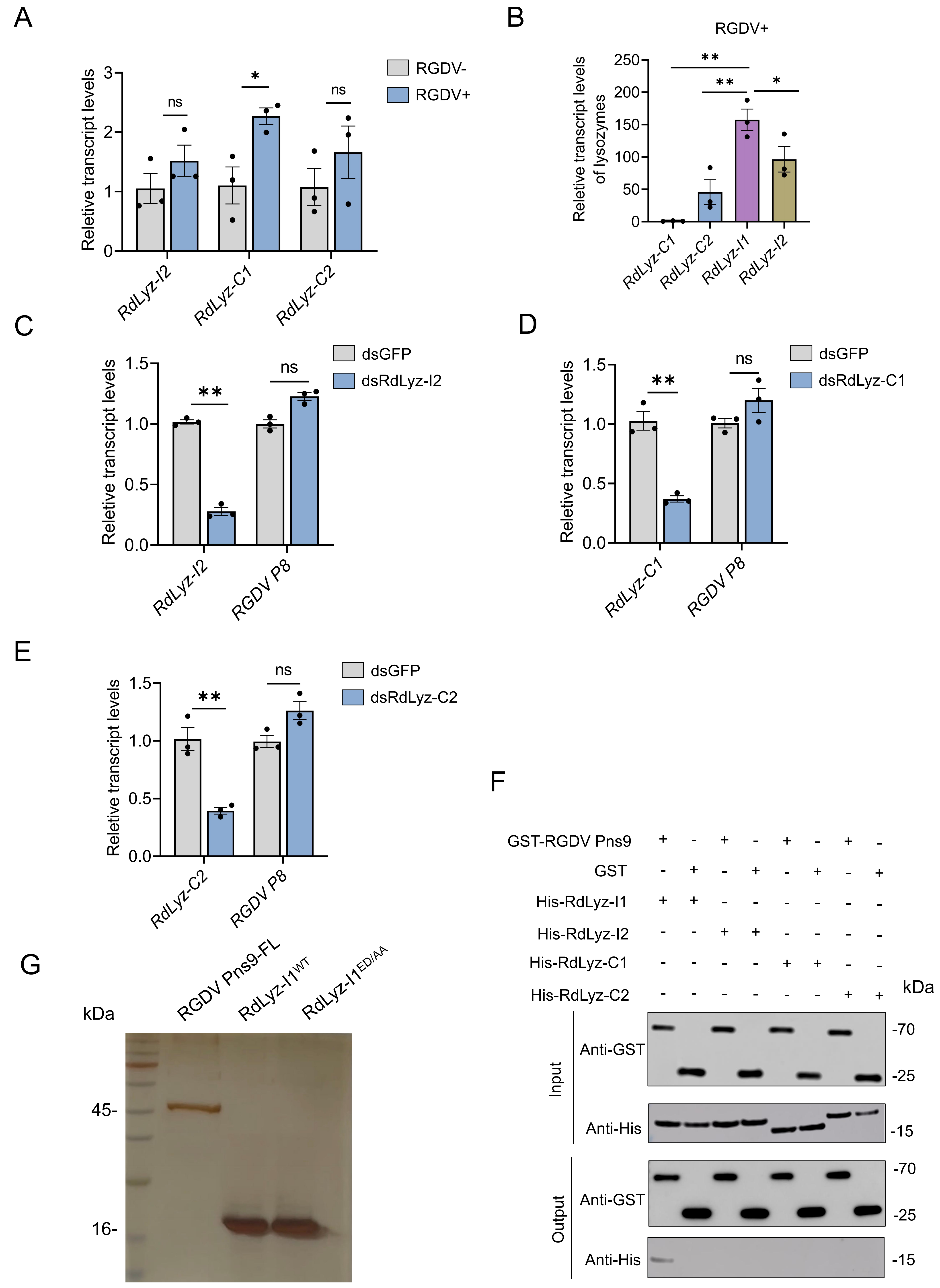


Figure S1. *R. dorsalis* *lysozyme* genes expression upon RGDV infection. A) RT-qPCR analysis showing the expression of *R. dorsalis Lyz-I2*, *Lyz-C1*, and *Lyz-C2* genes in nonviruliferous or viruliferous leafhoppers. RNA was extracted from 30 pooled leafhoppers per replicate. B) RT-qPCR analysis showing the expression of *R. dorsalis* four *lysozyme* genes in viruliferous leafhoppers. RNA was extracted from 30 pooled leafhoppers per replicate. C-E) Effects of *Lyz-I2* C), *Lyz-C1* D), and *Lyz-C2* E) knockdown on RGDV infection, as determined by RT-qPCR. RNA was extracted from 30 pooled viruliferous leafhoppers per replicate. Relative expression for *Lyz-I2*, *Lyz-C1*, *Lyz-C2* and RGDV *P8* are shown. Data in A), B), C), D), and E) are mean ± SEM (ns, not significant; *, *P* < 0.05; **, *P* < 0.01; Student’s *t*-test). All data represent three biological replicates. F) GST pull-down assays showing specific interaction between RGDV Pns9 and RdLyz-I1. No binding was detected between RGDV Pns9 and other *R. dorsalis* lysozymes (RdLyz-C1, RdLyz-C2, RdLyz-I2). Interacting proteins were detected using either anti-GST or anti-His antibodies. G) Silver staining of SDS-PAGE gel showing purified RGDV Pns9-FL, RdLyz-I1, and RdLyz-I1^ED/AA^ proteins.


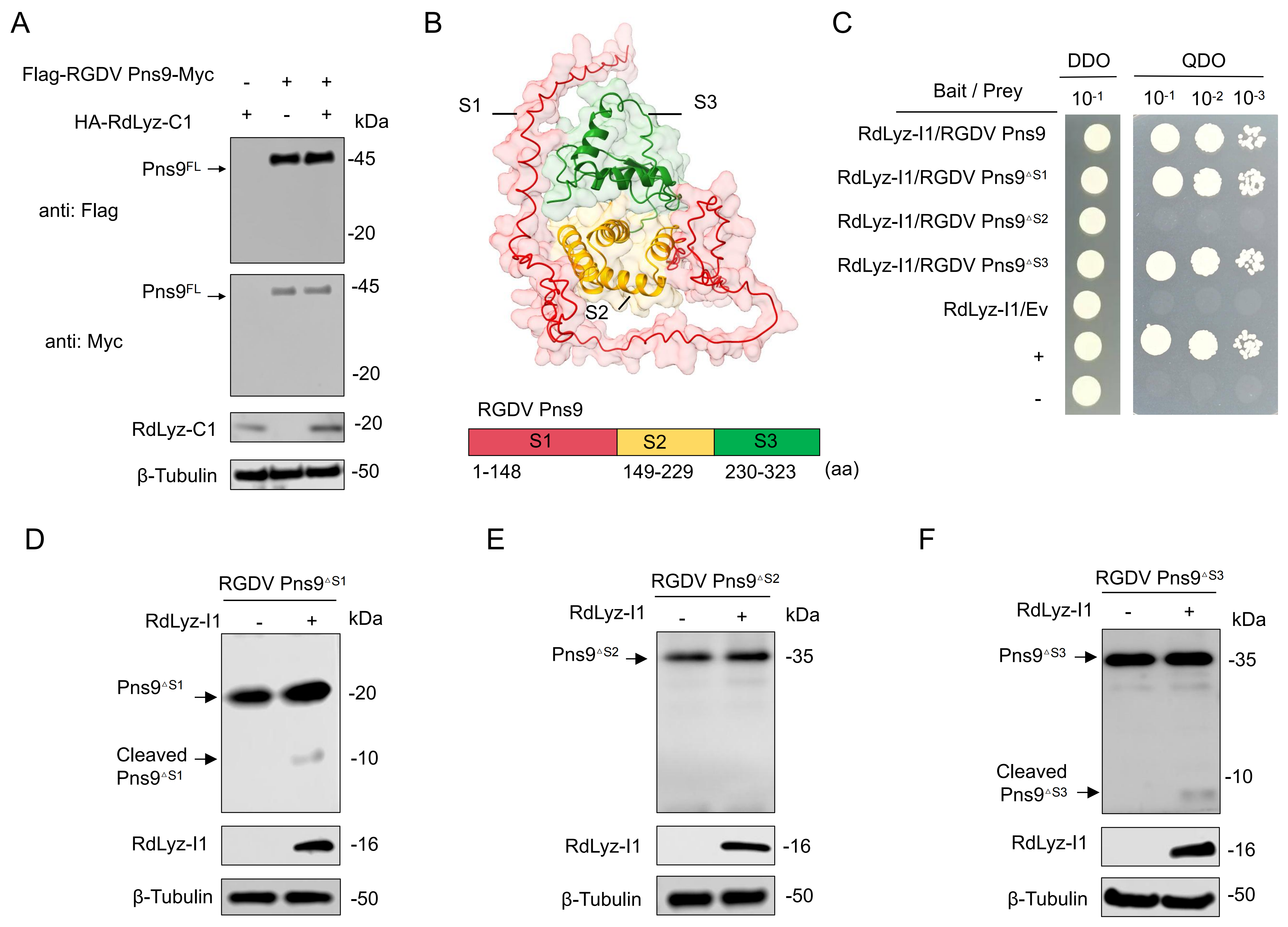


Figure S2. RdLyz-I1 binds to the RGDV Pns9 S2 region and cleaves it specifically. A) Western blot analysis of RGDV Pns9 cleavage and RdLyz-C1 expression in HEK-293T cells co-transfected for 24 h with 1.0 μg either of Flag-Pns9-Myc and HA-Lyz-C1 expression plasmids. Detection utilized anti-Myc, anti-Flag and anti-HA antibodies. B) Predicted structure of RGDV Pns9 generated using AlphaFold3. The three distinct regions of Pns9 are indicated. C) Y2H assays analysis of interactions between RdLyz-I1 and RGDV Pns9 or its deletion mutants (Pns9^△S1^, Pns9^△S2^, Pns9^△S3^). Transformants were plated on DDO (SD/-Trp-Leu) and QDO (SD/-Trp-Leu-His-Ade) media. Data are from one representative of three independent experiments. D-F) Western blot analysis of RGDV Pns9 cleavage and RdLyz-I1 expression in HEK-293T cells co-transfected for 24 h with 2.0 μg HA-RdLyz-I1 plasmids and 1.0 μg either of Pns9 deletion mutants Pns9^△S1^ D), Pns9^△S2^ E), or Pns9^△S3^ F) expression plasmids. Proteins were detected using anti-Flag and anti-HA antibodies.


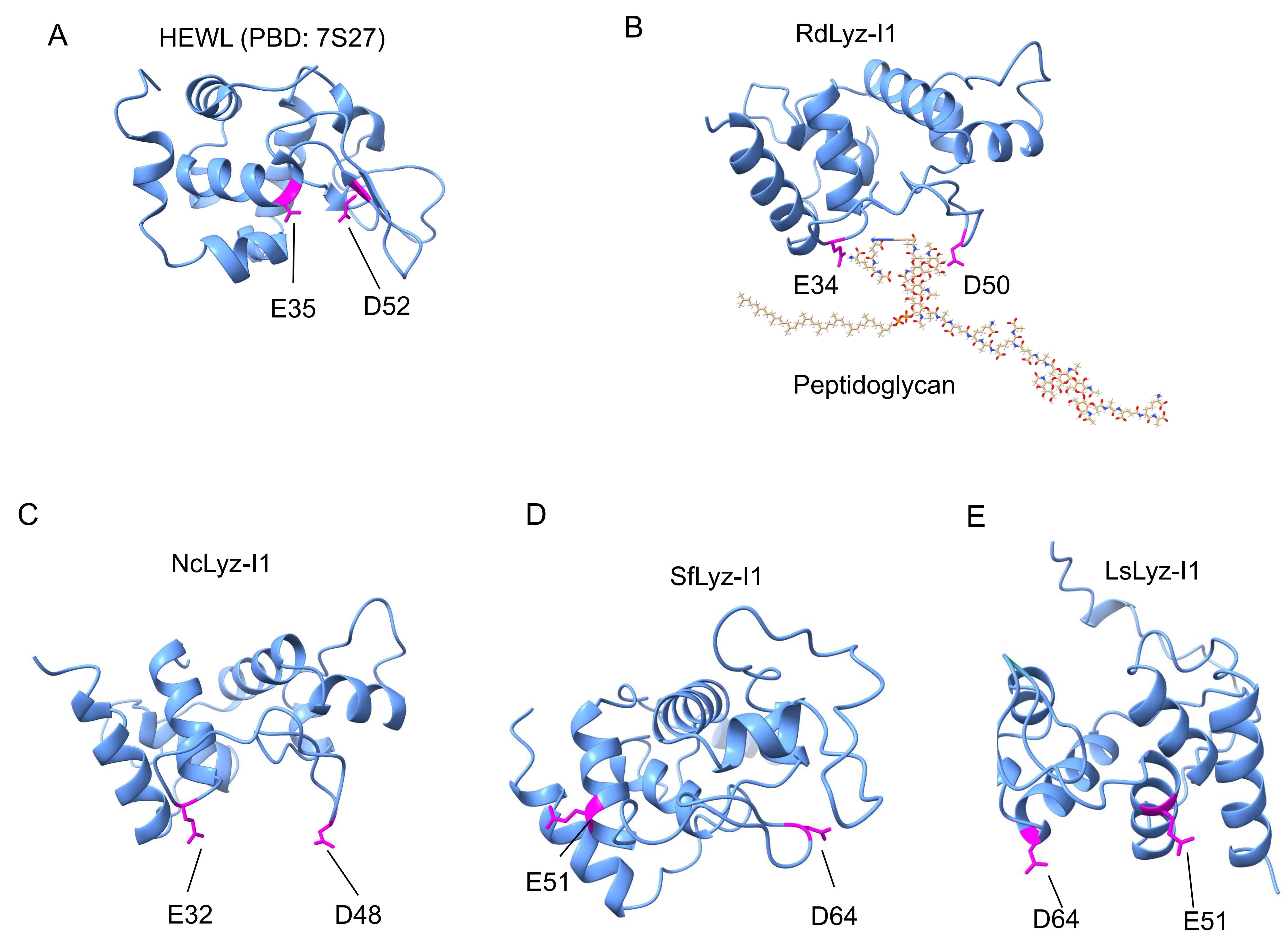


Figure S3. Structural and catalytic residues analysis of lysozymes. A) Structural predictions of hen egg-white lysozyme (HEWL) from AlphaFold3. B) Structural model of RdLyz-I1 in complex with peptidoglycan. The schematic illustrates the predicted binding mode between RdLyz-I1 and a fragment of bacterial peptidoglycan. Key catalytic residues E34 and D50 are highlighted and positioned near the glycosidic bond of the peptidoglycan strand, consistent with their proposed role in substrate hydrolysis. C-E) Structural predictions of NcLyz-I1 C), SfLyz-I1 D), and LsLyz-I1 E) from AlphaFold3. The conserved catalytic residues Glu (E) and Asp (D) are highlighted in dark and light purple.


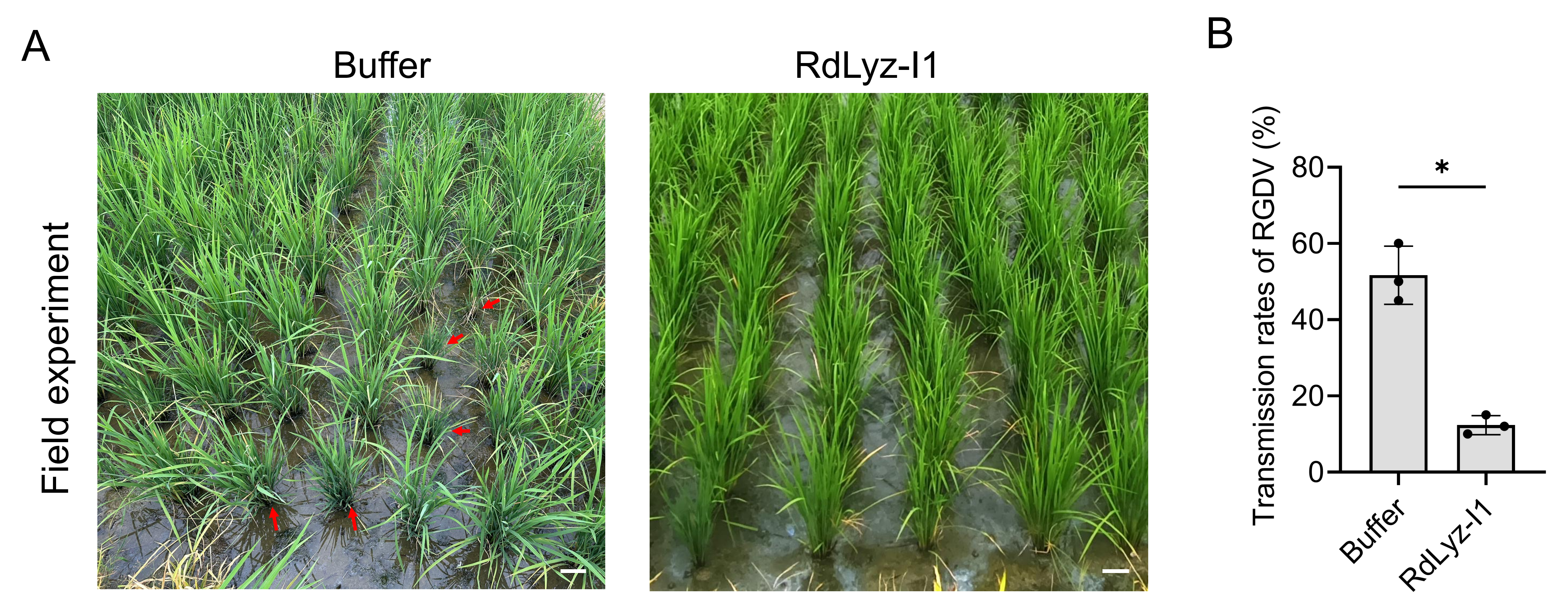


Figure S4. Field evaluation of RdLyz-I1-mediated resistance against rice virus transmission under natural conditions. A) Field experimental layout for assessing the protective efficacy of 0.5 mg/mL RdLyz-I1 under natural conditions. Rice plants were exposed to viruliferous leafhoppers in an open field environment. Red arrows indicate rice plants showing typical symptoms of gall dwarf disease at 40 days after initial treatment. Scale bars, 10 cm. B) Disease incidence in RdLyz-I1-treated and control groups. Plants received three exogenous applications of 0.5 mg/mL RdLyz-I1 or PBS buffer (control). Data represent mean ± SEM (n = 3 biological replicates; *, *P* < 0.05, Student’s *t*-test).


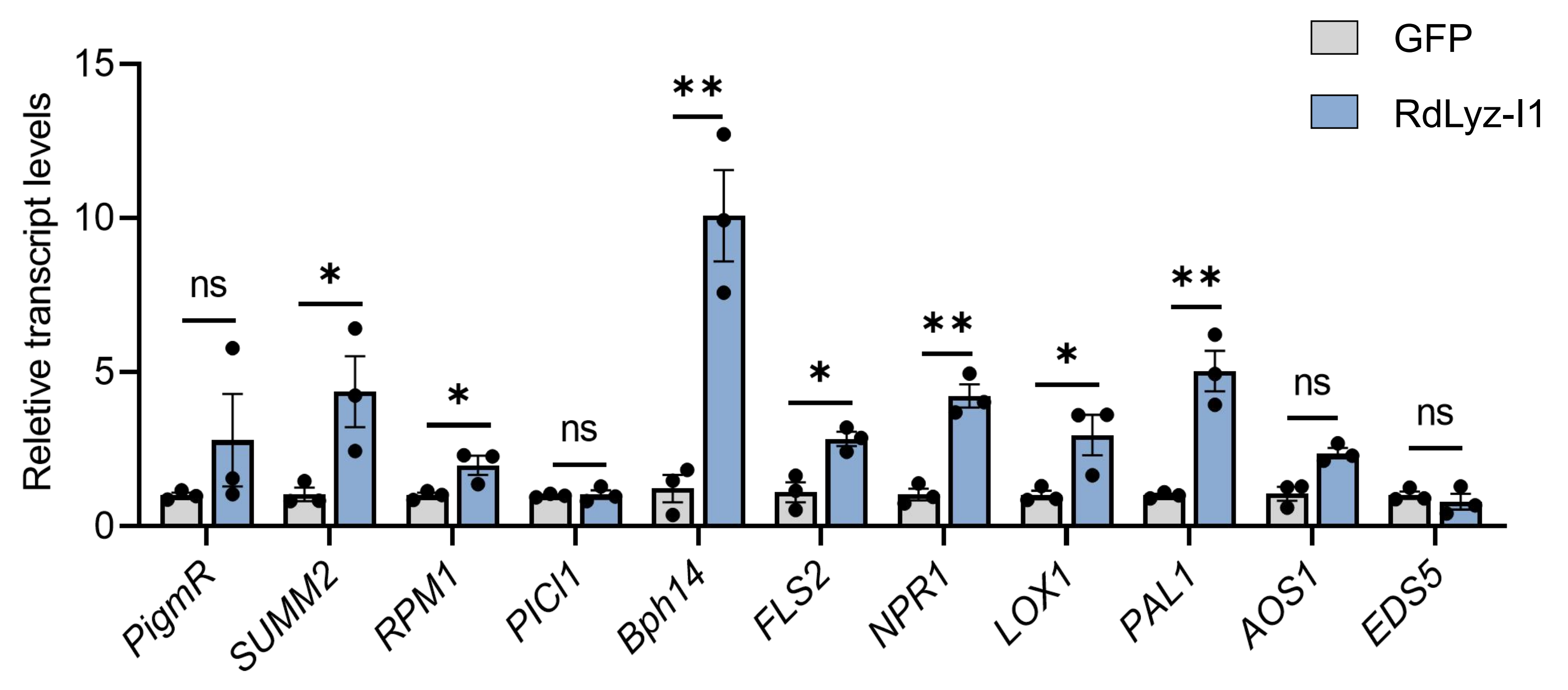


Figure S5. RT-qPCR analysis of immune-related genes expression in rice leaves after treatment with 0.5 mg/mL GFP or RdLyz-I1 protein. Leaves were collected from healthy rice seedlings (approximately 10 cm height) at 14 days post-treatment. Total RNA was extracted from 0.5 g leaf samples per biological replicate for subsequent analysis. Data are presented as mean ± SEM (ns, not significant; *, *P* < 0.05; **, *P* < 0.01; Student’s *t*-test).

**Table S1. Primers used in this study.**

| **Primer name** | **5'-3'** |
| --- | --- |
| F-RdLyz-I1-Q | TAGAAGGGTTGTTTCGCGCT |
| R-RdLyz-I1-Q | AGCTGGGACCTTCGTTGATG |
| F-RdLyz-I2-Q | GACCCTTCGGATGCCAGAAA |
| R-RdLyz-I2-Q | TGTCAAGCACTGCAACAACTG |
| F-RdLyz-C1-Q | GGTGATGGTAGCCACAGTCC |
| R-RdLyz-C1-Q | CGGCTGAGGTGTTGAACTCT |
| F-RdLyz-C2-Q | GTTGCTGGTAGGTGGTGTGT |
| R-RdLyz-C2-Q | TACCTGGGGAGGGACAACTC |
| F-RGDV P8-Q | TGAATGACCCAAGAACCCCG |
| R-RGDV P8-Q | ACGTTTGGCGCTACATCTGA |
| F-RdToll1-Q | CTCTCCCCCGAAGTCCAGTA |
| R-RdToll1-Q | CGGTCCACTCTGTAGATCGC |
| F-RdMyD88-Q | TCTGAGACCTTCTACTCGTCAA |
| R-RdMyD88-Q | GGTATTCCTCCAACAGTCCAAG |
| F-RdDorsal-Q | TTGTGGTCTCCTGTGTCA |
| R-RdDorsal-Q | GCCTCCTCAATATCCTTCTT |
| F-RdEF1-Q | CAGTGAGAGCCGTTTTGAG |
| R-RdEF1-Q | AGGGCATCTTGTCAGAGGGC |
| F-PigmR-Q | AGGAGAGCATCCGCCATCTA |
| R-PigmR-Q | AACAAAGTGCAGGTGTTCGC |
| F-SUMM2-Q | CTTGCAACTCTTGGTCGTGC |
| R-SUMM2-Q | TGGGATCTCATGAATGCGGG |
| F-RPM1-Q | TCCAGGTTGCAGGTTGATCC |
| R-RPM1-Q | TTTGGCATTGTGCCAGCTTC |
| F-PICI1-Q | GAACAGCGACTCGGAGAAGA |
| R-PICI1-Q | CTCGCCATAGACCTGGACG |
| F-Bph14-Q | TTAAGAGTTGCTGCCTGGAAT |
| R-Bph14-Q | GTGAGCATCCCAGCTTCTTCT |
| F-FLS2-Q | TGGCTGCTTTACTCGGATGG |
| R-FLS2-Q | GAAGGCCTTGGAGGGTTGTT |
| F-NPR1-Q | AGCTAGTCAGGATGCTGC |
| R-NPR1-Q | TTCGCCTCGCAGCAATGTGA |
| F-LOX1-Q | ATCCAGCTCCTAACGGAG |
| R-LOX1-Q | TGGTAGTCCAGGATGAAGA |
| F-PAL1-Q | AGAGCTCCGTCAAGAACTGC |
| R-PAL1-Q | TCTGCATGAGCGGGTAGTTG |
| F-AOS1-Q | TTCATGCCGTCCACCGAC |
| R-AOS1-Q | ACACCTCGCGGAACTTGG |
| F-EDS5-Q | ACCAACATTGGCATTCAG |
| R-EDS5-Q | TGCGATGACGATGGCTGT |
| F-OsActin-Q | TGTATGCCAGTGGTCGTACCA |
| R-OsActin-Q | CCAGCAAGGTCGAGACGAA |
| F-dsGFP | TAATACGACTCACTATAGGGCGAATGGTGAGCAAGGGCGAGG |
| R-dsGFP | TAATACGACTCACTATAGGGCGACTTGTACAGCTCGTCCATGCC |
| F-dsRdToll1 | TAATACGACTCACTATAGGGCGAATGCTCCAAATACAGTCCTACG |
| R-dsRdToll1 | TAATACGACTCACTATAGGGCGACATCTTGCATCCACTCAGTCTC |
| F-dsRdMyD88 | TAATACGACTCACTATAGGGCGATACGATGCGCTGCTGTTATACG |
| R-dsRdMyD88 | TAATACGACTCACTATAGGGCGATAACTTGACTGAAATCATTTTAG |
| F-dsRdDorsal | TAATACGACTCACTATAGGGCGAGCAGGTTCCATAGACTTGA |
| R-dsRdDorsal | TAATACGACTCACTATAGGGCGAGGTATCTCCGTGTTATCCAT |
| F-dsRdLyz-I1 | TAATACGACTCACTATAGGGCGAGCTACCTGGTGGGCTGCC |
| R-dsRdLyz-I1 | TAATACGACTCACTATAGGGCGAGTTCTCCCCGAACCGCAC |
| F-dsRdLyz-I2 | TAATACGACTCACTATAGGGCGACCAGTAGCTGTAACTTGA |
| R-dsRdLyz-I2 | TAATACGACTCACTATAGGGCGAGACACCTGGTGGCTCGGA |
| F-dsRdLyz-C1 | TAATACGACTCACTATAGGGCGACCTGTGGGCTCACCTGCG |
| R-dsRdLyz-C1 | TAATACGACTCACTATAGGGCGAAGAGTACTCGTCGAGGAA |
| F-dsRdLyz-C2 | TAATACGACTCACTATAGGGCGAGATCCCACCACAAGATGT |
| R-dsRdLyz-C2 | TAATACGACTCACTATAGGGCGACTCAGTATCCTCATCGTC |
| F-RdLyz-I1^△SP^ | ATGATCGACGACAGCTGCATCCACT |
| R-RdLyz-I1^△SP^ | TTAGTTCTCCCCGAACCGCA |
| F-RdLyz-I1^E34A^ | TCTGTGTgccATCAACGAAGGTCCCAGCTGTA |
| R-RdLyz-I1^E34A^ | CGTTGATggcACACAGACAGTGGATGCAGCTG |
| F-RdLyz-I1^D50A^ | ACgccGGAGTGTGTGGACCTTATGCCATCACC |
| R-RdLyz-I1^D50A^ | TCCACACACTCCggcGTGGCAGCCCACCAGGTAG |
| F-RdLyz-I1^△L2^ | ATGATCGACGACAGCTGCATCCACT |
| R-RdLyz-I1^△L2^ | GCAGTCTTGTAGCGCGTTCCT |
| F-RdLyz-I2^△SP^ | ATGATTTGTAGTATCTGGTTT |
| R-RdLyz-I2^△SP^ | TACCAATTGTACATACATAA |
| F-RdLyz-C1^△SP^ | AGGGTGTACGACCGCTGCGAGC |
| R-RdLyz-C1^△SP^ | TCAAGAGTACTCGTCGAGGA |
| F-RdLyz-C2^△SP^ | AAGGTGTTCGACAGGTGTG |
| R-RdLyz-C2^△SP^ | TCACTCAGTATCCTCATCGTC |
| F-RGDV Pns9 | ATGTTTACATCTTCTGCAGCCAAG |
| R-RGDV Pns9 | TTAAGGTTCTTCCACGTCTCGTAA |
| F-RGDV Pns9^T179A^ | AAACGTCgccAAAGAGCTGAACGACCCGAACG |
| R-RGDV Pns9^T179A^ | GCTCTTTggcGACGTTTAAGACTGCCATCATAAATT |
| F-RGDV Pns9^K180A^ | TAAACGTCACTgccGAGCTGAACGACCCGAACG |
| R-RGDV Pns9^K180A^ | CTCggcAGTGACGTTTAAGACTGCCATCATAA |
| F-RGDV Pns9^△S1^ | AAATATCCGTCCATGTTGACCA |
| R-RGDV Pns9^△S1^ | TTAAGGTTCTTCCACGTCTCGTAA |
| F1-RGDV Pns9^△S2^ | ATGTTTACATCTTCTGCAGCCAAG |
| F2-RGDV Pns9^△S2^ | tgtagctatcGAGCCATGGTCAGATGGAGTATC |
| R1-RGDV Pns9^△S2^ | accatggctcGATAGCTACAAAGCTATCTCCTCTTTCT |
| R2-RGDV Pns9^△S2^ | TTAAGGTTCTTCCACGTCTCGTAA |
| F-RGDV Pns9^△S3^ | ATGTTTACATCTTCTGCAGCCAAG |
| R-RGDV Pns9^△S3^ | TTCCGCCAGACGCATCAGACTTGC |
| F-NcLyz-I1 | ATGATCGACGACGCCTGCATCAAAT |
| R-NcLyz-I1 | CATACGGTTTGGAGGGGACAAGTAG |
| F-NcLyz-I1^E32A^ | CTGTGTgccGCTATCAACGAGGGCCCGATATG |
| R-NcLyz-I1^E32A^ | TTGATAGCggcACACAGGCATTTGATGCAGGC |
| F-NcLyz-I1^D48A^ | CTGCCACgccGGGGTGTGTGGGCCTTACGCAA |
| R-NcLyz-I1^D48A^ | ACACCCCggcGTGGCAGCCCTCCTGGTAGTTG |
| F-RDV P7 | ATGTCTGCGATTGTAGGCCTCT |
| R-RDV P7 | TAAAGCTGACAGCTTTTGTATGAGA |
| F-RDV P7^K238A^ | TCCCATCgccGAATTATTCTCGATGGGCTCTGG |
| R-RDV P7^K238A^ | ATAATTCggcGATGGGAGTATTCAAGCACGCA |
| F-SfLyz-I1^△SP^ | ATGCAATGGGCTTACGATGTCGCTCG |
| R-SfLyz-I1^△SP^ | TCAGCTTCTCATTTTGCTGATAC |
| F-SfLyz-I1^E51A^ | CATTTGTgccACCATTGATTGCACTATGATCAATACA |
| R-SfLyz-I1^E51A^ | CAATGGTggcACAAATGCAGTCCAGGCAGTCG |
| F-SfLyz-I1^D/A^ | TGTAAGGGTgccTACTGCGGACCATTTTCCATC |
| R-SfLyz-I1^D/A^ | CAGTAggcACCCTTACATGTATTGATCATAGTGCA |
| F-SRBSDV Pns9 | ATGGCAGACCTAGAGCGTAGAACG |
| R-SRBSDV Pns9 | AACGTCCAATTTAAGTGAAGAAAAGT |
| F-SRBSDV Pns9^K244A^ | CAGTTCCTGAAgccAAAATCAATCACATTTATTATCAACTTGC |
| R-SRBSDV Pns9^K244A^ | TTTggcTTCAGGAACTGTAGTACCGACTAGATATT |
| F-LsLyz-I1^△SP^ | ATGGAAAGGGATAACGACGTTGCTCG |
| R-LsLyz-I1^△SP^ | TCAGCTTTTCATTTTACTGAGACAC |
| F-LsLyz-I1^E51A^ | GCATTTACgccACCATAAATTGCACTGGAATTAGTAAA |
| R-LsLyz-I1^E51A^ | TATGGTggcGTAAATGCAGTTCAGACATTCATCAG |
| F-LsLyz-I1^D64A^ | ATGCATGGGTgccATCTGTGGACCGTTCTCCATTAA |
| R-LsLyz-I1^D64A^ | AGATggcACCCATGCATTTACTAATTCCAGTG |
| F-RBSDV Pns9 | ATGGCAGACCAAGAGCGGA |
| R-RBSDV Pns9 | AACGTCCAATTTCAAGGAAGAAA |
| F-RBSDV Pns9^K244A^ | GTTCCAAATgccGAAGTTAATCACATTTTTTACCAGCTG |
| R-RBSDV Pns9^K244A^ | ACTTCggcATTTGGAACTCTTGTTCCGATGAG |
| F-RdDorsal | ATGGCTGCATCCGTTATGGAGTTGT |
| R-RdDorsal | TCATTTGACAGTTTGGGAAAGACCT |
| F-RdLyz-I1-pHis2 | gactcactatagggcgaattcGATTATCAAAATATCCCGATTTTCAA |
| F-RdLyz-I1-pHis2 | gcgtgagctccccgggaattcCCGCGATGTTTTTGCGGC |
| F-RGDV P8-PCR | GTGGCTAGATCGTCAGCCA |
| R-RGDV P8-PCR | TTATTGCATGTTAAACTTACCCTC |
